# Clinical protein science in translational medicine targeting malignant melanoma

**DOI:** 10.1007/s10565-019-09468-6

**Published:** 2019-03-21

**Authors:** Jeovanis Gil, Lazaro Hiram Betancourt, Indira Pla, Aniel Sanchez, Roger Appelqvist, Tasso Miliotis, Magdalena Kuras, Henriette Oskolas, Yonghyo Kim, Zsolt Horvath, Jonatan Eriksson, Ethan Berge, Elisabeth Burestedt, Göran Jönsson, Bo Baldetorp, Christian Ingvar, Håkan Olsson, Lotta Lundgren, Peter Horvatovich, Jimmy Rodriguez Murillo, Yutaka Sugihara, Charlotte Welinder, Elisabet Wieslander, Boram Lee, Henrik Lindberg, Krzysztof Pawłowski, Ho Jeong Kwon, Viktoria Doma, Jozsef Timar, Sarolta Karpati, A. Marcell Szasz, István Balázs Németh, Toshihide Nishimura, Garry Corthals, Melinda Rezeli, Beatrice Knudsen, Johan Malm, György Marko-Varga

**Affiliations:** 1 Clinical Protein Science & Imaging, Biomedical Centre, Department of Biomedical Engineering, Lund University, BMC D13, 221 84 Lund, Sweden; 2 Section for Clinical Chemistry, Department of Translational Medicine, Lund University, Skåne University Hospital Malmö, 205 02 Malmö, Sweden; 3 Translational Science, Cardiovascular Renal and Metabolism, IMED Biotech Unit, AstraZeneca, Gothenburg, Sweden; 4 Division of Oncology and Pathology, Department of Clinical Sciences Lund, Lund University, 221 85 Lund, Sweden; 5 Department of Surgery, Clinical Sciences, Lund University, SUS, Lund, Sweden; 6 Department of Haematology, Oncology and Radiation Physics, Skåne University Hospital, Lund, Sweden; 7 Department of Analytical Biochemistry, Faculty of Science and Engineering, University of Groningen, Groningen, The Netherlands; 8 Department of Experimental Design and Bioinformatics, Faculty of Agriculture and Biology, Warsaw University of Life Sciences, Warsaw, Poland; 9 Chemical Genomics Global Research Lab, Department of Biotechnology, College of Life Science and Biotechnology, Yonsei University, Seoul, Republic of Korea; 10 Second Department of Pathology, Semmelweis University, Budapest, Hungary; 11 Department of Dermatology, Semmelweis University, Budapest, Hungary; 12 Cancer Center, Semmelweis University, Budapest, 1083 Hungary; 13 MTA-TTK Momentum Oncology Biomarker Research Group, Hungarian Academy of Sciences, Budapest, 1117 Hungary; 14 Department of Dermatology and Allergology, University of Szeged, Szeged, H-6720 Hungary; 15 Clinical Translational Medicine Informatics, St. Marianna University School of Medicine, Kawasaki, Kanagawa Japan; 16 Department of Surgery, Tokyo Medical University, 6-7-1 Nishishinjiku Shinjiku-ku, Tokyo, Japan; 17 Van’t Hoff Institute of Molecular Sciences, 1090 GS Amsterdam, The Netherlands; 18 Biomedical Sciences and Pathology, Cedars-Sinai Medical Center, Los Angeles, CA USA

**Keywords:** Malignant melanoma, Translational medicine, Clinical proteomics, Post-translational modifications, Cancer moonshot

## Abstract

Melanoma of the skin is the sixth most common type of cancer in Europe and accounts for 3.4% of all diagnosed cancers. More alarming is the degree of recurrence that occurs with approximately 20% of patients lethally relapsing following treatment. Malignant melanoma is a highly aggressive skin cancer and metastases rapidly extend to the regional lymph nodes (stage 3) and to distal organs (stage 4). Targeted oncotherapy is one of the standard treatment for progressive stage 4 melanoma, and BRAF inhibitors (e.g. vemurafenib, dabrafenib) combined with MEK inhibitor (e.g. trametinib) can effectively counter BRAFV600E-mutated melanomas. Compared to conventional chemotherapy, targeted BRAFV600E inhibition achieves a significantly higher response rate. After a period of cancer control, however, most responsive patients develop resistance to the therapy and lethal progression. The many underlying factors potentially causing resistance to BRAF inhibitors have been extensively studied. Nevertheless, the remaining unsolved clinical questions necessitate alternative research approaches to address the molecular mechanisms underlying metastatic and treatment-resistant melanoma. In broader terms, proteomics can address clinical questions far beyond the reach of genomics, by measuring, i.e. the relative abundance of protein products, post-translational modifications (PTMs), protein localisation, turnover, protein interactions and protein function. More specifically, proteomic analysis of body fluids and tissues in a given medical and clinical setting can aid in the identification of cancer biomarkers and novel therapeutic targets. Achieving this goal requires the development of a robust and reproducible clinical proteomic platform that encompasses automated biobanking of patient samples, tissue sectioning and histological examination, efficient protein extraction, enzymatic digestion, mass spectrometry–based quantitative protein analysis by label-free or labelling technologies and/or enrichment of peptides with specific PTMs. By combining data from, e.g. phosphoproteomics and acetylomics, the protein expression profiles of different melanoma stages can provide a solid framework for understanding the biology and progression of the disease. When complemented by proteogenomics, customised protein sequence databases generated from patient-specific genomic and transcriptomic data aid in interpreting clinical proteomic biomarker data to provide a deeper and more comprehensive molecular characterisation of cellular functions underlying disease progression. In parallel to a streamlined, patient-centric, clinical proteomic pipeline, mass spectrometry–based imaging can aid in interrogating the spatial distribution of drugs and drug metabolites within tissues at single-cell resolution. These developments are an important advancement in studying drug action and efficacy in vivo and will aid in the development of more effective and safer strategies for the treatment of melanoma. A collaborative effort of gargantuan proportions between academia and healthcare professionals has led to the initiation, establishment and development of a cutting-edge cancer research centre with a specialisation in melanoma and lung cancer. The primary research focus of the European Cancer Moonshot Lund Center is to understand the impact that drugs have on cancer at an individualised and personalised level. Simultaneously, the centre increases awareness of the relentless battle against cancer and attracts global interest in the exceptional research performed at the centre.

## Introduction

Since ancient times, tumorous diseases have been known and were recognised by the Greeks as an imbalance of body fluids and an accumulation of ‘black bile’ (Falzone et al. 2018; Karpozilos and Pavlidis 2004). Melanomas mainly present as a dark-coloured-to-black mass, are visible to the eye and represent an apparent disease. Although exposure to ultraviolet (UV) radiation and rare genetic susceptibility within some ethnic groups are associated with the development and progression of melanoma, very little is known about the aetiology of this tumour (Dimitriou et al. 2018). The estimated worldwide incidence of melanoma varies between 15th–19th places amongst the most common cancers according to the GLOBOCAN database, whilst in Europe rises to the sixth position (Ferlay et al. 2015; IARC 2018; Leonardi et al. 2018). The most frequently affected primary sites are the torso in men and the limbs in women.

**Fig. 1:**
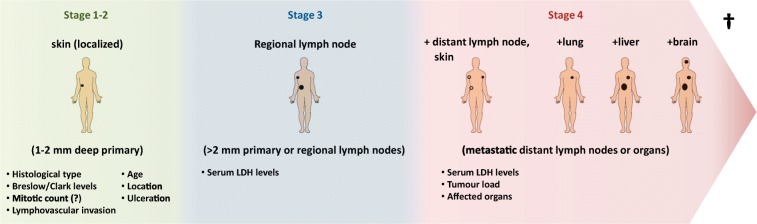
Progression of melanoma with prognostic factors at each stage. Tumour thickness is a key determinant in predicting prognostic outcome. With time, metastases develop and infiltrate multiple organs

As with other types of tumours, the TNM classification (where T refers to tumour size, N to lymph node involvement, M to metastatic spread) and staging of melanoma are still the gold standard prognostic factors for this malignancy (Breslow 1970; Keohane et al. 2018). Practical prognosis of melanoma is based on the depth of invasion (Breslow scale) into the skin (Breslow 1970; Keohane et al. 2018). Initially, a level of 0.76 was considered the threshold for early-stage melanoma, and surgery with an adequate margin of resection is curative (stages 1 and 2). Melanoma, however, tends to recur in approximately 10–20% of patients and metastases extend to the regional lymph nodes (stage 3) and to distal organs (stage 4) (Falzone et al. 2018). During the progression of melanoma (Fig. 1), the rate at which the disease advances increases, i.e. the 5-year survival rate for localised melanoma is 98.4%, for regionally metastatic, 63.6%, and for distant metastatic, 22.5%. The multiple visceral and brain metastases are primarily responsible for the death of patients (Sandru et al. 2014).

Recently, the World Health Organisation (WHO) introduced the 2nd melanoma pathology classification. This is now considered the new global standard (2017). Additionally, WHO provides examples of typical images of tumour morphologies. Sequencing and BRAF inhibitor therapy changed the course of the disease for metastatic patients, as BRAF mutation is one of the key targetable genetic aberrations that occurs in melanomas (Chapman et al. 2011). Further DNA alterations have been described and medication is available also in combination to target key pathways. As a general trend, however, the metastatic melanoma (MetM) escapes from this blockage and in almost all instances progresses (Hauschild et al. 2012; Chapman et al. 2011; Chiappetta et al. 2015; Larkin et al. 2015; Tringali et al. 2014). There is room for improvement in understanding tumour biology, predicting prognosis and developing more effective therapies.

In the development of malignant melanoma, molecular alterations and protein modifications are responsible for the acquisition of a metastatic phenotype. In this regard, clinical protein science, or clinical ‘proteomics’, links to functional genomics by providing a role and function to specific protein(s) in a given medical and clinical setting. In this respect, the uniquely broad versatility of proteomics, with dedicated applications to biological mass spectrometry (MS), is a requirement for achieving reliable results within many areas of life science. No other technology has such a diverse array of methods and protocols, and solid MS experiments are often linked to functional conclusions.

Through collaborative efforts between academia and modern healthcare, a cutting-edge cancer research centre with a specialisation in melanoma and lung cancer has been established. Our focus is on the impact of drugs on cancer and includes:

- Verification of disease mechanisms and disease staging
- Mode-of-drug action
- Unravelling the complexity of cancer
- Functional confirmation of the disease link
- Determining the site(s) of post-translational modifications

## Disease presentation in melanoma patients

Malignant melanoma (MM) is the most aggressive type of skin cancer and develops from pigment-containing cells known as melanocytes (Dimitriou et al. 2018). These pigment-forming cells migrate from the neural crest to colonise the ectoderm during foetal life. Thus, the cells occur primarily in the skin; however, such cells also exist on the genitalia, in the mouth and in the eye (Table 1). During the past few decades, the incidence of melanoma has been continuously increasing together with no significant improvement in mortality. Recent epidemiological study stated melanoma within the top three cancers with the largest increase in incidence (39% from 2006 to 2016) (Falzone et al. 2018; Fitzmaurice et al. 2018). Due to improved screening and surveillance programs, there has been a dramatic increase in thin melanomas. The frequency of thick melanomas, however, has remained stable (Linos et al. 2009). Intrinsic aetiology of melanoma includes predisposition of melanoma-associated genes, Fitzpatrick skin type, familiar atypical mole syndrome and giant congenital nevi (Rigel 2010). Amongst the extrinsic factors, exposure to UV light is still considered the primary environmental driver of the genesis of melanoma (El Ghissassi et al. 2009). Additionally, men often have a higher occurrence on the back, whilst with women, the most common occurrence is on the legs (Glazer et al. 2017). It is known that the primary cause of melanoma is exposure to UV light. This risk increases when combined with low levels of skin pigment, a compromised immune system and other genetic factors (Erdei and Torres 2010).

### Clinical phenotypes of primary melanoma

In the vast majority of cases, patients present with the primary melanoma lesion on the skin. However, other types of melanomas such as subungual, mucosal and ocular can also occur.

#### Main clinical types of primary melanoma

Superficial spreading melanoma (SSM) on sun-exposed skin is responsible for more than half of the melanoma cases that primarily affect middle-aged patients. SSM appears as a dark macule or plaque that has usually become altered in appearance according to the ABCDE rules. According to these melanoma signs, ABCDE classification is based on:

A— Asymmetry
B— Border irregularity: notched border
C— Colour variegation: red, white, blue, dark brown
D— Diameter of the melanoma
E— Evolution of moles

Regression is not unusual and leads to polychromatic (amelanotic or even dark bluish dermal melanotic) areas. Other areas within the atypical plaque can be more papular or nodular indicating secondary vertical growth of the SSM. BRAFV600E is the main genetic driver but is not specifically the mutation for SSM (Bauer et al. 2011). Nodular melanoma (NM) occurs at an older age than SSM but is less frequent. NM can present anywhere on the body and presents as a rapidly growing nodule with secondary ulceration. NM occurs via any of the main driver gene mutations (Broekaert et al. 2010).

The UV-driven melanoma, lentigo maligna melanoma (LMM), occurs on sun-exposed areas of elderly people: primarily the face, back and extremities. LMM correlates strongly with UV-induced signature mutations (Curtin et al. 2005). Although LMM is the most indolent form of melanoma, in long-lasting, neglected cases, a vertical growth phase also occurs. Acral lentiginous melanoma (ALM) presents as variable pigmented plaques on the palms, soles and subungual areas and affects middle-aged to elderly patients. ALM initially shows flat lentiginous spreading; however, continuous trauma and loss of compliance often induces a vertical growth phase. Mutation of c-kit is a characteristic finding for ALM and also for mucosal melanomas (Curtin et al. 2006). The desmoplastic melanoma is considered an UV-induced melanoma that appears on sun-exposed areas of elderly people, and presents as a firm dermal mass mimicking a soft tissue tumour. Clinical and histopathological diagnosis can thus be challenging. Desmoplastic melanoma tends to progress to haematogenous metastases rather than lymphatic spread (Murali et al. 2010), and is associated with a very high UV mutation rate and signature prone to immunotherapy (Eroglu et al. 2018). Metastatic melanoma transformed from blue nevus (malignant blue nevus) are bluish-coloured dermal plaques or nodules with secondary ulceration or haemorrhage. Similar to ocular melanomas, these have a characteristic GNAQ mutation affecting G protein–coupled receptors (Arkenau et al. 2011).

**Fig. 2:**
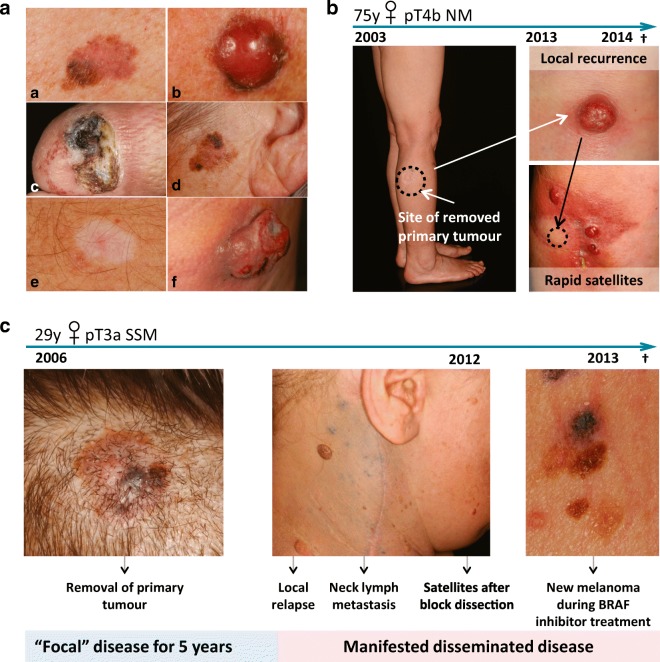
**(**A) Representative clinical pictures of superficial spreading melanoma (a), nodular melanoma (b), acral melanoma (c) and lentigo maligna melanoma (d). Regression (e) exhibits a whitish, non-specific macule without a palpable tumour, whereas the bulky tumour phase (f) shows a large, ulcerated, rapidly growing nodule. (B) Clinical images of minimal residual disease. Case study of a 75-year-old woman who had had a tumour removed from the primary site over a decade earlier. After 10 years of dormancy, the tumour recurred locally. Upon removal of the recurrent tumour, several weeks later satellite tumours developed. (C) Clinical images of a young female with a completely excised scalp lesion. After 5 years, local relapse followed by lymph node metastases and satellite tumour formation occurred. During targeted BRAFV600E therapy, the patient developed resistance and new terminal melanomas developed (all from the database of the Onco-dermatological Unit, Department of Dermatology and Allergology, University of Szeged)

Other infrequent types of melanoma such as childhood melanoma can occur as rapidly growing atypical nodules within a congenital nevus. Spitzoid melanoma together with BAP-1 pathway–inactivated melanoma (Busam 2013) may display as firm skin-coloured, verrucous or polypous nodules mimicking a conventional wart or skin tag (Fig. 2A).

#### To be or not to be? Changed growth in primary melanoma (regression, bulky tumour)

Regression is a pathological term defined as a disappearance of dermal and junctional melanoma cells that are replaced by fibrosis, permeation of inflammatory infiltrate and melanophages, together with neovascularisation (Emanuel et al. 2008). Clinically, melanoma regression is observed as a thinned, whitish or bluish area during the clinical course of melanoma. The significance of regression is still debated as there are *pro* and *contra* data concerning the role played in melanoma progression, recurrence or survival (Kaur et al. 2007; Søndergaard 1985). Nevertheless, there are clinical observations that have shown the appearance of loco-regional metastases during the regression of the primary melanoma defined as smouldering phenomenon (Piérard et al. 2012). Indeed, the cellular composition of regression includes tumour-associated macrophages and cancer-associated fibroblasts that are known to play a crucial role in tumour promotion. Conversely, tumour-infiltrating lymphocytes undoubtedly have anti-tumoural effects within the melanoma-derived microenvironment (Ziani et al. 2017). Along these lines, regression may be considered a paradoxical one step forward to loco-regional and distant metastases.

Bulky tumour or the tumourigenic phase shows as a rapid change as an increase of the primary melanoma. In the majority of cases, this is why patients are referred to a dermatologist. Tumour growth can be based on sequential melanoma genesis followed by SSM, ALM or LMM in a vertical growth phase. De novo nodular melanomas, however, can occur within weeks in the same manner as pronounced ulcerated nodules. The increased thickness of these tumours requires urgent treatment which can be accompanied by a good response; otherwise, the prognosis is dismal (Chapman et al. 2015).

### Features of progression and metastasis

#### Local recurrence

After removal of a primary melanoma, local recurrence is used as an independent prognostic factor and is usually indicative of a worst prognosis. In cases where a broad excision and complete removal of the primary melanoma was performed, recurrent satellite melanomas can be explained by the reactivation of dormant tumour–derived cells in the peri-tumoural stroma due to uncertain stimuli (Wong et al. 2005).

#### Loco-regional (lymph node) disease

Lymph node metastasis is due to the lymphogenous spreading of melanoma cells via peri-tumoural lymphatic vessels to the regional lymph nodes. The first target lymph node of melanoma cells is defined as the sentinel lymph node, which should be removed and processed by histopathology. If the pathological stage of primary melanoma reaches at least pT1b (presence of ulceration, or thickness is more than 0.8 mm according to AJCC 8th edition Gershenwald 2017 (Gershenwald et al. 2017)), sentinel lymph node biopsy (SLNB) is routinely performed, which has a prognostic value and may also indicate further treatment (block dissection, or adjuvant therapy). An internationally validated nomogram (Pasquali et al. 2011) to predict possible involvement of sentinel lymph node was developed based on clinic-pathological factors such as age, location of tumour, tumour thickness and presence of ulceration (Wong et al. 2005). Loco-regional melanoma metastasis indicates at least stage 3 disease.

#### Distant (haematogenous) metastases

As a consequence of disseminated melanoma, visceral (e.g. lungs, liver, spleen, kidneys) or brain metastases occur in advanced stage 4 and have a poor prognosis (Gershenwald et al. 2017).

#### Circulating melanoma cells

Blood-borne metastatic melanoma cells are not only observed in disseminated stage 4 disease. These have also been detected in early-stage loco-regional or minimal residual disease. Thus, detection thereof is crucial for enhanced screening and melanoma diagnostics (Scaini et al. 2019).

### Open questions on special courses and progression of melanoma

#### Special patient courses shed light on minimal residual disease of melanoma

According to clinical staging, patients often show a tumour-free state for several years following complete removal by wide excision of the primary melanoma. Unfortunately, rapid local recurrence, and/or loco-regional or disseminated metastases, often occur. The phenomenon of late progression after a disease-free state has been attributed to early dissemination of dormant, clinically non-apparent melanoma cells before removal of the primary tumour (Röcken 2010).

The fact that melanoma cells disseminate at an early stage (when the primary tumour exists) but do not manifest suggests the existence of a minimal residual disease. This interesting phenomenon of melanoma should direct oncological perception towards an awareness that dormant metastases probably already exist even in clinically non-apparent cases. The questions that now arise should ask ‘which patient’, and ‘when’ and ‘how’ melanoma metastases manifest from this minimal residual disease. This hypothesis is also relevant for two other peculiar forms of melanoma: extremely late metastasis formation (more than 10 years after the removal of the primary tumour) and donor-derived melanoma metastases in recipients after many years following transplantation (Strauss and Thomas 2010; Tsao et al. 1997).

Another debated question is whether a relationship exists between tissue healing and subsequent progression of melanoma. This is because the inflammatory environment induced by melanoma is similar to the wound-healing microenvironment. After the removal of the primary melanoma, hidden tumour cells can lead to an early local recurrence adjacent to the surgical scar. Neither the exact pathways nor evidence-based clinical data are known to support this hypothesis, and only scattered case reports are available on this topic (Tseng and Leong 2011).

The genomic, proteomic and other omic characterisation of the appropriated cohort of samples can provide the data to address all the questions regarding the progression and different outcomes in melanoma. As ultimately proteins are the effectors of most the cellular functions, the analysis of expression, post-translational modifications and mutations thereof are extremely valuable to understand the biology of melanoma. The patterns observed at the protein level including the post-translational modifications can be correlated to differences in the progression of the disease, resistance to a particular treatment or the preference to metastasise to a specific organ. In addition, the integration of several omic approaches and clinical data has the potential to revolutionise the way cancer is treated today.

A case report on a 75-year-old female highlights the importance of minimal residual disease. The disease remained in a latent phase for 10 years before the sudden rapid progression of melanoma (Fig. 2B). The patient presented with ‘high-risk’ thick nodular melanoma on her right leg but had not shown any clinically apparent dissemination for a decade. Ten years after the complete removal by wide excision of the primary melanoma, a local recurrent lump had appeared. The tumour was excised; however, weeks following surgery, rapid new satellite tumours developed. The patient died within weeks because of the rapid dissemination of the metastatic disease. These rapid, multiple recurrent and metastatic cases are prone to targeted therapy (Falzone et al. 2018; Robert et al. 2015).

#### Clinical aspects of oncotargeting: phenotype switch of melanoma

To date, targeted oncotherapy is the standard treatment for rapidly progressive stage 4 disease. BRAF inhibitors (e.g. vemurafenib, dabrafenib) combined with MEK inhibitor (e.g. trametinib) effectively attack BRAFV600E-mutated melanomas (Falzone et al. 2018; Robert et al. 2015). Compared to conventional chemotherapy, this treatment strategy does result in a significantly higher response rate in reducing the bulky masses. Thus, patients in a preterminal state are prevented from a rapid death (Flaherty et al. 2012). After a period of progression-free disease, however, most responsive patients develop resistance to the therapy and lethally progress (Pimiento et al. 2013).

Shown in Fig. 2C are the clinical images from a case report on a young female. The patient presented with high-risk superficial spreading melanoma on the scalp that had been completely excised. Her past case history included chronic lymphocytic leukaemia. There was no sign of clinical dissemination for 5 years. Local recurrence, rapid spreading to the neck lymph nodes, cutaneous satellites and visceral progression developed after block dissection. Targeted therapy was initiated as the melanoma was BRAFV600E positive; however, new tumours were identified on the back region during BRAF inhibition. The patient showed resistance to targeted therapy and passed away after rapid progression of the melanoma.

The many underlying factors behind developed resistance to BRAF inhibitors have been extensively studied. These include reactivation of the mitogen-activated kinase (MAPK) pathway and activation of wild-type BRAF, and epigenetic changes (Pimiento et al. 2013). Recent interest has focused on phenotype switching of melanoma. Acquisition of a low microphthalmia-associated factor MITF state together with activation of epithelial-mesenchymal transition (EMT) can transform melanoma cells to a highly invasive, dedifferentiated and therapy-resistant phenotype with cancer cell plasticity (Kemper et al. 2014). Melanoma cells can gain EMT state by the downregulating of E-cadherin together with the upregulating of N-cadherin and osteonectin pathways (Alonso et al. 2007). Repressors of E-cadherin are SLUG and ZEB1 transcription factors (Wels et al. 2011). Furthermore, dedifferentiation of melanocytes can be driven by the loss of ZEB2 transcription factor. For example, as decreased ZEB2 expression was associated with significantly reduced metastasis-free survival in melanoma patients (Caramel et al. 2013). Similarly, MITF not just plays a crucial role in the differentiation state, but its loss leads to the metastatic phenotype of melanoma (Hoek et al. 2008). This change from proliferative into invasive state of melanoma is regulated by decreased LEF1 and increased TCF4 expression (Eichhoff et al. 2011). As well as signalling pathways are considered, in addition of the well-known MAPK and PI3K regulation, receptor tyrosine kinase (RTK) and TGF-β signalling are also involved in the phenotype switching and subsequent metastatic phenotype of melanoma (Kemper et al. 2014; Li et al. 2015). These widespread changes can be responsible for the clonal evolution of heterogeneous melanoma tissue and also for the clinically apparent evolution of the disease in response to the iatrogenic ‘medical’ environment.

#### Unsolved clinical questions call for proteomic solutions

During the past few decades, the prognostic factors for melanoma have unfortunately remained unaltered. There is still histopathological staging that focuses primarily on tumour thickness, and clinical staging that is an estimate of the clinical behaviour of primary melanoma (Gershenwald et al. 2017). Although the main driver genes (BRAF, NRAS, C-KIT, NF, GNAQ) have been discovered, these markers cannot act as individual indicators for every melanoma case. Therefore, there is a fundamental need for novel prognostic biomarkers. Similarly, individualised prognosis and personalised therapeutic predictors are of prime importance. Amongst the driver genes, BRAF is regarded as the main predictor for targeted therapy. The BRAFV600E mutation, however, is identified at the genetic level but known targeted therapies act on proteins. In addition, there is still a lack of evidence concerning the influence of tissue heterogeneity in melanoma tissue on the effect and outcome of targeted therapy. Therefore, whether the BRAFV600E mutation is homogenously or heterogeneously translated to the level of the mutated proteins in melanoma tissue requires further characterisation. Moreover, the lack of any insights into the development of protein resistance after targeted therapy calls for proteomic approaches for personalised medicine.

## Pathological characterisation of melanoma

### Cancer tissue heterogeneity

Genomic instability results in the occurrence of heterogeneous events in the DNA. This is considered a hallmark of cancer and provides selective advantage for the survival of tumour cells either in a dormant phase or as a rapidly progressing disease (Jin et al. 2018). Tissue heterogeneity in melanoma is key to the survival and evolutionary versatility of the disease (Hwang et al. 2018), and some clinical studies have shown that reduced heterogeneity is linked to a longer survival of the cancer patients (Gara et al. 2018; Hanahan and Weinberg 2011; Szász et al. 2017). To some extent, heterogeneity exists in all types of malignant tumours. This is already evident with the diversity of cells that exist within a neoplasm and the proteomic profiles thereof.

In routine diagnostics, attempts have been made to characterise the tissue in more detail. This was previously mentioned for melanoma treatment; whereby serial sections of a sentinel lymph node are taken to detect cancer cells in the context of the surrounding heterogeneous tissue. Here, size is an important aspect as the largest dimension of the tumour must be captured and recorded. This then acts as a guide towards further treatment, e.g. regional block dissection to harvest more lymph nodes. In diagnostics, deeper levels of histological samples are often requested to support and aid pathological diagnostics. In every consecutive tissue section, there are changes in the arrangement and composition of the cells. These changes can range from minimal to substantial morphological heterogeneity through all levels of a tumour. By extrapolation, such cellular heterogeneity implies that a tumour and the surrounding tissues are comprised of a broad and diverse range of proteins (Welinder et al. 2017).

### Digital pathology and machine learning/artificial intelligence

Digital pathology is a novel platform that can be used to obtain spatial information from tissue architecture (Cooper et al. 2015; Madabhushi and Lee 2016). It is best applied to tissues after fixing and stabilising with formalin. Therefore, digital pathology can be applied to patient tissues that have been processed in the clinical pathology laboratory. After embedding in paraffin, the tissues are sectioned, placed on glass slides and stained with haematoxylin and eosin (H&E) for microscopic examination. Stained slides can also be digitised at high resolution for analysis. As an alternative to H&E, tissues can be stained by immunohistochemistry and immunofluorescent antibody detection. Due to the high resolution of digital images, protein expression and protein complex formation can be measured in subcellular compartments. A multiplexed antibody or in situ RNA hybridisation format can be used to measure up to 40–60 proteins per slide with technologies such as multiplexed fluorescence microscopy (MxIF) (Gerdes et al. 2013), imaging mass cytometry Tissue-CyTOF® (Giesen et al. 2014), digital spatial profiling (DSP) using NanoString Technology or co-detection by imaging (CODEX) (Goltsev et al. 2018). In a colorectal cancer study, by using MxIF, Gerdes et al. (2013) were able to map the signal transduction patterns of the kinase mTORC1 signalling. Measuring the phosphorylated targets of mTORC1, 4E-BP1 and RPS6, they could provide important clues regarding the mechanism of regulation of this pathway. In this sense, in theory, any proteoform or combination of proteoforms can be measured in individual cells.

Machine-learning algorithms that quantitate specific, predetermined patterns in images can be used to obtain data from protein expression and from cellular and tissue organisation. Alternatively, with deep-learning convolutional neural networks (CNN)/artificial intelligence, computers can be trained to identify patterns that distinguish subgroups of cancers, differing in prognosis or treatment response. Computers can also be trained to identify patterns in images in an unbiased manner and convert images into numerical data sets that capture spatial relationships with tissue structures (Madabhushi and Lee 2016). As a result, digital pathology data complements molecular data sets that are generated from tissue lysates. Altogether, digital pathology and machine learning provide novel opportunities for biomarker development. These quantitative imaging biomarkers can be integrated with molecular data and clinical variables to predict prognosis and treatment responses in patients.

An important application of digital pathology is the analysis of the immune infiltrate in melanoma. Several recent papers describe new methods to profile the magnitude, composition and activity associated with the spatial configuration of the tumour immune response. A recent paper used a deep learning/artificial intelligence approach to identify patterns of lymphocyte infiltration in tumours (Saltz et al. 2018). The authors applied a convolutional autoencoder to boost a CNN that was trained to recognise individual lymphocytes. For the first time, a CNN a.k.a. ‘computational stain’ was sufficiently accurate and efficient to count tumour-infiltrating lymphocytes (TILs) in cancer tissues from 4759 subjects and across 13 cancer types. Digital TIL numbers were correlated with molecular data to reveal associations with survival, tumour subtypes and immune profiles. The H&E images used for the study were procured by the Cancer Genome Atlas (TCGA) consortium. TCGA generated separate molecular profiling data for subcutaneous skin and uveal melanoma (Akbani et al. 2015; Robertson et al. 2017). These melanoma subtypes differ in the mechanism of cancer development and progression and with every case an H&E stained slide representative of tumour histology was included. In addition to unique molecular profiles, cutaneous and uveal melanomas differed in immune cell infiltration. On average, the immune infiltrate in uveal melanoma was sparse, except for a fraction of uveal melanomas with poor prognosis that displayed an extensive immune infiltrate (Van Raamsdonk et al. 2010; Robertson et al. 2017). Interestingly, this T cell infiltration, which consists of activated cytotoxic T cells and macrophages, has no effect on survival (de Lange et al. 2018). In contrast to uveal melanoma, skin cutaneous melanoma displays, as expected, one of the highest leukocyte fractions amongst 30 cancer types that were profiled by the TCGA consortium (Thorsson et al. 2018). A cluster analysis based on the spatial configuration of TILs identified four structural patterns and the cluster count separated good from poor prognosis melanoma subgroups (Saltz et al. 2018).

In addition to immune cells, other cell types can also be profiled in the tumour microenvironment using digital pathology and machine learning. For example, the vasculature is amenable to digital analysis (Ing et al. 2017). Microvessel density, lymphatic density and vascular invasion correlated with BRAF mutation status, suggesting a relationship between aggressive behaviour and vascular morphometric parameters (Aung et al. 2015). Since vascular organisation can be imaged through non-invasive methods, it could assist in the diagnosis of melanoma (Massi et al. 2002) and potentially also provide a pre-surgical assessment of tumour stage.

Most recently, several studies demonstrated a road of algorithmic pathology towards the clinic through applications that are directly related to patient care. In regular pathology practice, deep-learning algorithms using CNNs have the potential to provide a virtual second opinion and improve the efficiency of dermatopathologists (Olsen et al. 2018). In one study, the Google Inception v4 CNN was trained for detection of melanomas and the diagnostic performance was assessed with an international group of 58 dermatopathologists. Most were outperformed by the computer diagnostic tool (Haenssle et al. 2018). A machine-learning approach was also used prior to surgery to predict the risk of melanoma with promising results that approach the sensitivity and specificity of diagnostic evaluations by expert physicians (Gareau et al. 2017).

Altogether, recent advances in digital pathology, machine-learning and deep-learning CNNs represent disruptive technologies that are well situated to change the future practice of pathology. These methods have the potential to standardise the quality of pathological diagnoses, improve the efficiency of pathologists and assist in personalised treatment decisions. In particular, digital pathology can be applied to measure patterns of TILs that capture the anti-tumour activity of the immune system at the interface of the tumour. Digital data can also be integrated with genomic and proteomic data in prediction models of patient outcomes. Lastly, training computers to assist with patient stratification provides a cost-effective path to bring precision medicine to a broad range of communities and across larger populations.

## Biobanking and sample preservation

A fully integrated large-scale biobank infrastructure has been built at the Biomedical Centre in Lund. The centre provides storage space for preserving biological material (tissue and blood), processing and analysis of collected samples and sample shipment to scientific partners for clinical projects/collaborations. There is a fully automated platform and workflow where > 1000 sample tubes are processed per day. Robots tend to both blood fractions and tissues that are stored at − 80 °C (Fehniger et al. 2013; Malm et al. 2013, 2015, 2018; Marko-Varga et al. 2012b).

The Biomedical Centre acts as a hub with multiple clinical centres participating in this initiative, and tissue and blood samples are received into the melanoma biobank from all over the world. The centre was developed to generate and build large-scale biostorage archives of patient melanoma samples. These are then combined with histopathological expertise to characterise the patient tumours. This large-scale clinical sample processing enterprise was initiated with the aim of creating high-end histopathology indexing with database computational power and proteogenomic analysis. Subsequently, the biobank at Lund has become an important resource in global clinical research (Fehniger et al. 2013; Malm et al. 2013, 2015, 2018; Marko-Varga et al. 2012b). Several national health programs are now being initiated with the aim of also building large-scale biobank storage and populating these with a wealth of high-quality patient samples. In our cancer R&D activities, the samples in the biobanks and the data derived from these are aiding in deepening our understanding of disease presentation. This information drives research towards ‘Big Data’ proteogenomics and mass spectrometry imaging studies.

## Proteomics

Proteomics is a highly promising field to aid in the identification of cancer biomarkers and novel therapeutic targets. Proteomics is defined as the characterisation of proteins encoded by the genome of a given organism at a given time in a given state (Aebersold et al. 2018; Wasinger et al. 1995; Wilkins et al. 1996). The core principles of proteomics lie in the ability to perform sensitive analyses on a complex mixture of proteins and peptides. Proteomics can address challenges beyond the reach of genomics, i.e. relative abundance of the protein products, PTMs, protein localisation, turnover, protein interactions and protein function. The proteomic analysis of body fluids and tissues can be a valuable asset in the search for diagnostic and prognostic biomarkers.

**Fig. 3:**
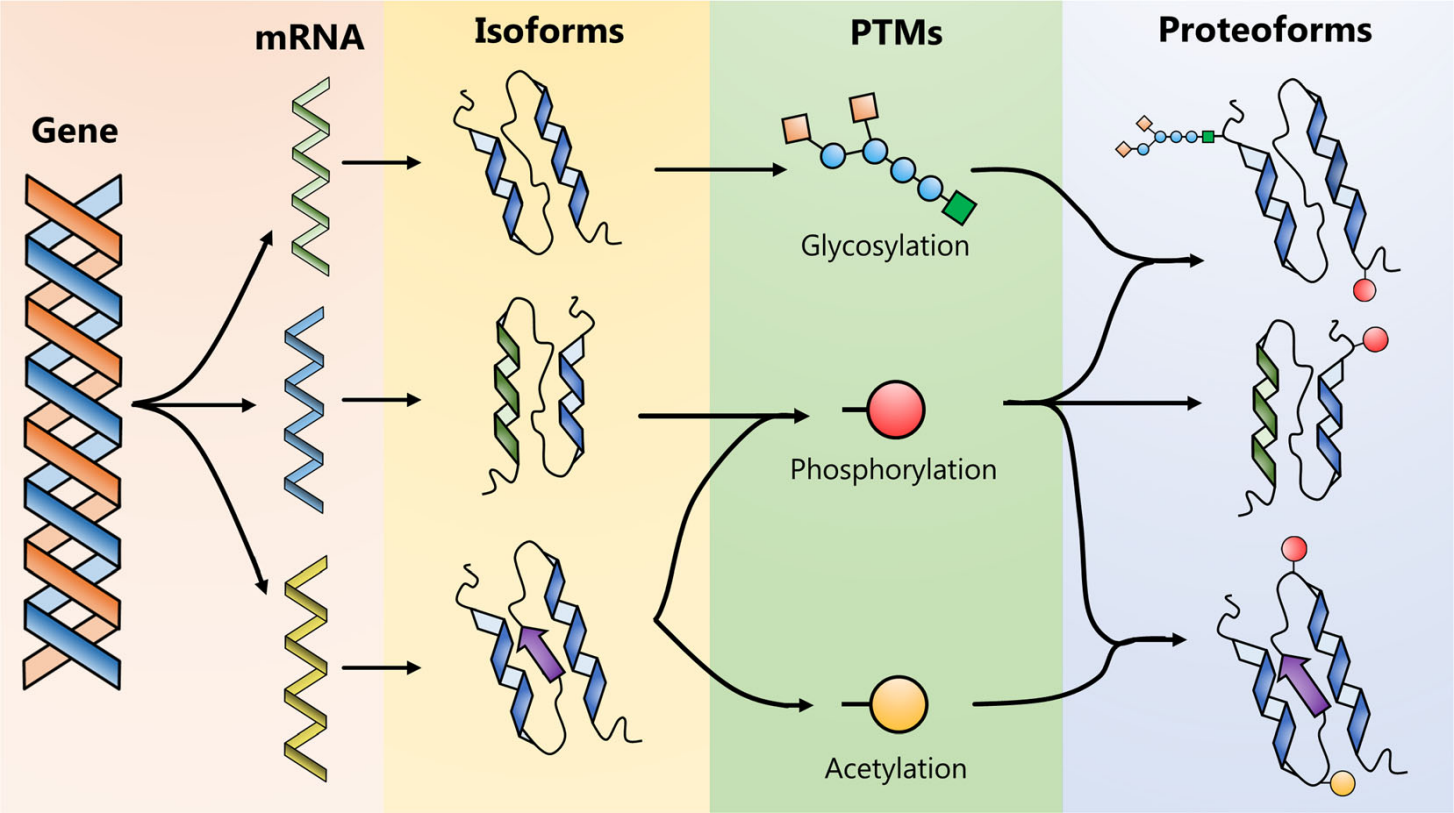
Schematic illustration of proteoforms formed by gene coded regions, undergoing post-translational modification

As a consequence of the human genome project, the number of protein-coding genes is now estimated at 20,377. According to the Human Proteome Project from the Human Proteome Organisation, these can be divided into five classes depending on the type of protein evidence (PE): PE1 (17,487, 85.8%) proteins identified by the highest stringency criteria including data from mass spectrometry (MS) analyses and antibody identification; PE2 (1728, 8.2%) by expressed mRNA transcripts; PE3 (515, 2.5%) by sequence similarity; PE4 (76, 0.4%) by in silico prediction; PE5 (571, 2.8%) representing proteins whose existence is uncertain (HPP, NextPro Release 2018-09-03). Many genes are transcribed as splice variants. When this is taken into consideration, the number of human proteins increases to 42,384. In addition, human proteins also undergo post-translational modification that strongly influences function and/or activity. There is a wide and diverse array of PTMs including modifications such as glycosylation, phosphorylation and acetylation (Fig. 3). Ultimately, such PTMs give rise to many hundreds of thousands of additional protein variants (Aebersold et al. 2018).

### Clinical proteomic pipeline

**Fig. 4:**
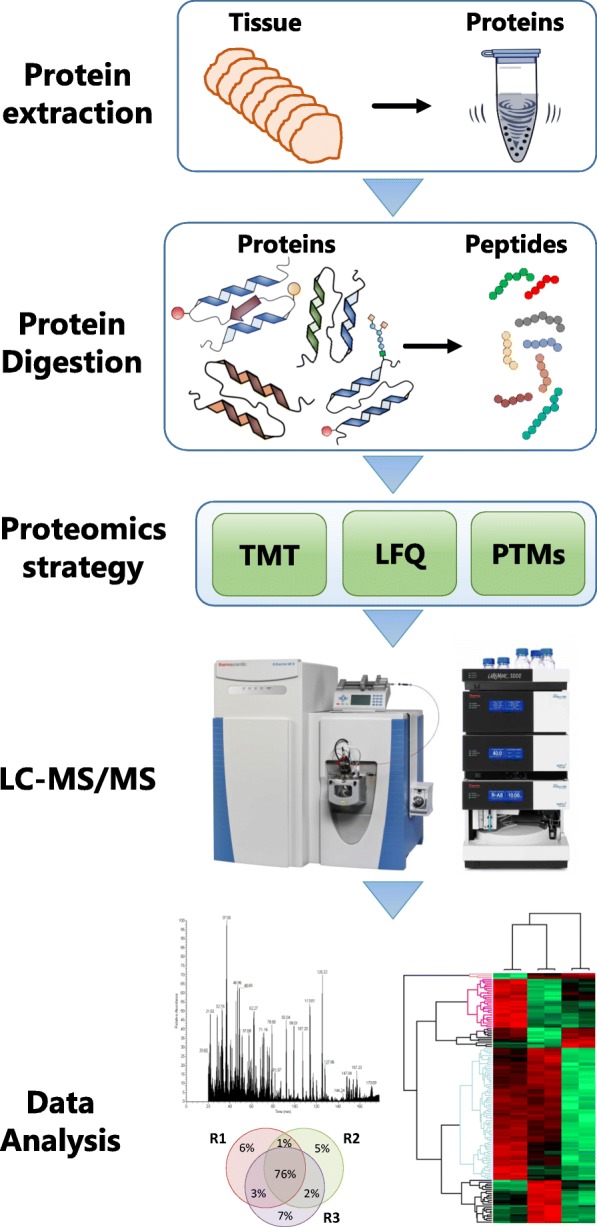
Clinical proteomic workflow. Tissue sections are processed to extract the proteins. These are digested and analysed by LC-MS/MS. Peptides are identified and quantitated via labelling approaches or by label-free methods. Peptides with specific PTMs can be enriched and also analysed by LC-MS/MS.

Shown in Fig. 4 is an overview of the different steps involved in current clinical proteomic workflows/pipelines. This includes tissue sectioning and histological examination, protein extraction from selected metastatic melanoma tissues and enzymatic digestion. Depending on the clinical question and the samples under investigation, these steps are then followed by such approaches as quantitative analysis based on label-free or labelling technologies, e.g. tandem mass tag (TMT) multiplexing and peptide fractionation, or enrichment of peptides with specific PTMs. Regardless of the decision-making process, complex peptide mixtures are injected onto a sensitive and high-resolution LC-MS/MS system, i.e. a nano-high-performance liquid chromatography (nHPLC) instrument coupled to a mass spectrometer. Peptides are separated by reversed-phase fractionation, detected by mass spectrometry (MS) and sequenced by tandem mass spectrometry (MS/MS). All data generated is then analysed to identify and quantitate the peptides and proteins.

**Fig. 5:**
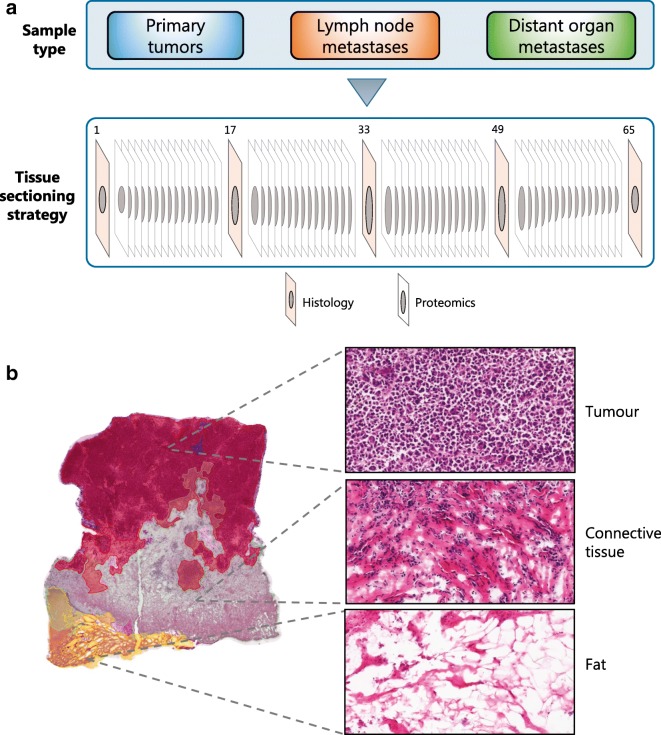
**a**, **b** Sample processing strategy for deep analysis of the melanoma proteome by mass spectrometry. Three different types of solid samples are stored in the biobank from melanoma patients: primary tumours, lymph nodes and distant organ metastases. Samples selected for analysis were cryo-sectioned. Fifteen slices are used for MS analysis and one section is prepared for histology to determine the tumour cell content and the percentage of other tissues that are present. The MM slices are prepared for quantitative proteomics

To maximise our knowledge, a cryo-sectioning strategy was implemented (Fig. 5). With this approach, histological images of all samples are recorded every 10–15 slices. This is of major importance, because within a tumour sample, the composition can vary significantly at different levels in terms of tumour cell content, presence of necrosis, infiltration of immunological cells and connective tissue content. In addition, from sliced tissues, the protein extraction is maximised without the need to macerate the sample. Each tissue section has a thickness of 10 μm and, on average, 15 sections of melanoma tumour sample weigh 7.8 mg, ranging from 5 to 10 mg. In these samples, the protein content and the number of proteins can vary depending on the composition. Overall, the protein recovery from melanoma samples is approximately 12% of the total weight of the tissue.

In the context of translational medicine where large cohorts are essential, the automation of the different steps of sample preparation can result in a significant improvement in reproducibility and a reduction in the intrinsic variability of manual procedures. The incorporation of automated steps during sample preparation maximises the likelihood of discovering new and meaningful findings. The implementation of automation at the protein extraction and enzymatic digestion steps has shown an effective increase in throughput and a marked reduction in experimental variability (Kuras et al. 2019).

The protein extraction protocol plays a fundamental role within any proteomic pipeline, as it provides the starting material for all subsequent steps during sample processing. Previous studies have indicated that this step is the major source of variation in proteomics (Piehowski et al. 2013). On the proteomic platform developed for MM, the Bioruptor plus (model UCD-300) is utilised for protein extraction. This device uses temperature-controlled ultrasound technology for highly-efficient disruption and homogenisation of the tissues with minimal operator participation. Up to 12 samples can be simultaneously processed, thus increasing throughput and reducing processing time. For the lysis buffer, urea or SDS/DTT extraction solutions are used (Wiśniewski et al. 2009). In our hands, both solutions provided similar results in terms of the number of identified proteins; however, protein yield is higher with SDS/DTT. This is particularly true for samples with a very low tumour cell density and/or high content of connective tissue.

Usually in proteomics, protein extraction is followed by the enzymatic digestion of proteins, in most cases with trypsin alone or combined with other enzyme(s). Via LC-MS/MS analysis, generated peptides are central to both identifying and quantitating the proteins. For large cohorts of samples, this step is performed in the automated micro-chromatographic platform Bravo AssayMAP, in order to ensure the reproducibility of the hydrolysis. The Bravo AssayMAP is useful in a variety of procedures, from enzymatic digestion and peptide purification/concentration to specific affinity purification steps such as phosphopeptide enrichment (see below). Digestion of urea-containing MM lysates was easily implemented on the Bravo AssayMAP for MM samples. For these samples, digestion can be performed after simple dilution because trypsin tolerates moderate amounts of this chaotropic agent. Conversely, the low tolerance of trypsin to the presence of SDS requires a buffer exchange step before digestion, which can then be performed in the presence of sodium deoxycholate (SDC). SDC is a trypsin-compatible detergent usually used in proteomics (Gil et al. 2017; Lin et al. 2008).

Even though the majority of the proteomic approaches involve a protein extraction step and enzymatic digestion, different strategies provide different outcomes. This is particularly the case for quantitative proteomics, where a variety of methods are available. These methods are divided in two major groups, those based on differential isotopic labelling of each sample, such as TMT, and those using label-free approaches. In addition, the study of post-translational modifications usually requires specific methods. For example, to characterise the phosphorylation status of the proteins, the proteomic workflow is adapted to include a phosphopeptide enrichment step. The workflow can be readily adapted or modified to provide data on specific PTMs, and protein–protein or drug–protein interactions. For quantitative proteomics, phosphoproteomics and acetylomics of MM samples, different approaches were implemented based on isotopic labelling (TMT 11-plex) or label-free analyses.

#### TMT 11-plex labelling for quantitative proteomics

**Fig. 6:**
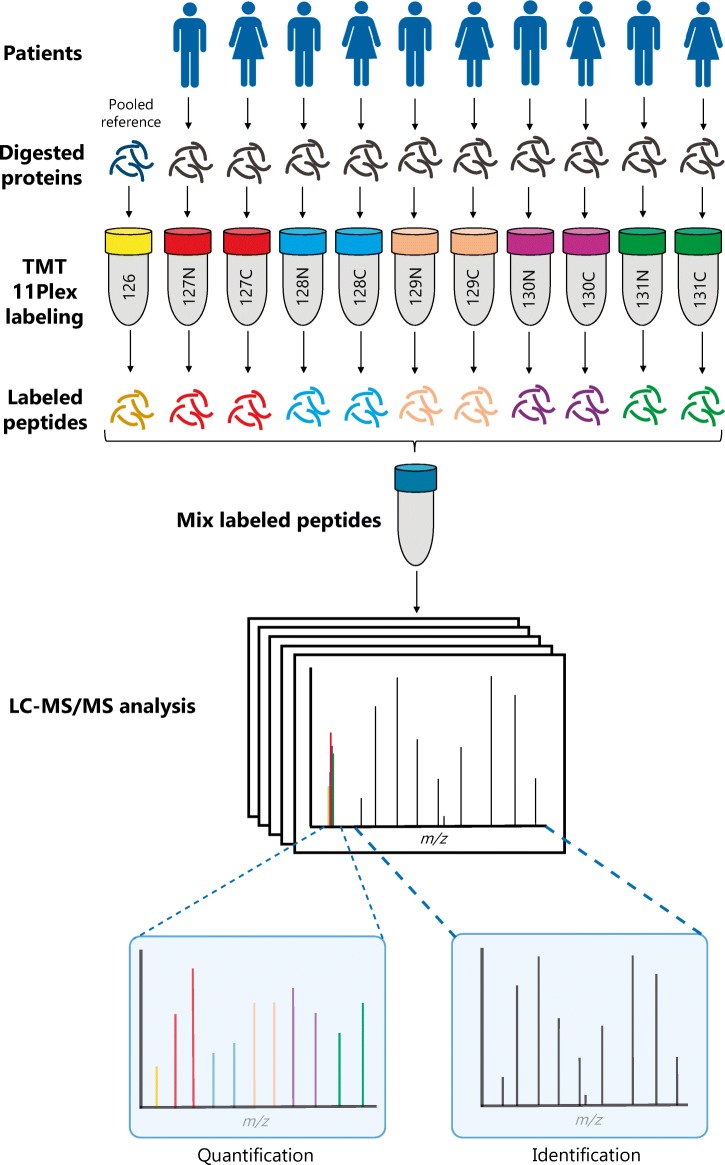
The principles of TMT multiplex labelling. Tissue samples from ten patients are processed and enzymatically digested. The resultant peptides are individually labelled with the TMT 10-plex reagents and all 10 samples are mixed together with a ‘standard’ comprised of a pool of peptides from all patients. The combined 11 labelled samples are analysed by LC-MS/MS to identify and quantitate the peptides/proteins

TMT 11-plex is a powerful technology that enables the simultaneous relative quantitation of proteins by mass spectrometry in up to 11 different biological samples. There are 11 different mass-tagging reagents with the same nominal mass and chemical structure. Each are composed of an amine-reactive NHS-ester group, a spacer arm and a mass reporter (Fig. 6). For every sample, a unique reporter ion mass signal in the low mass region of the MS/MS spectrum is used to measure relative protein expression levels following peptide fragmentation. When analysing tumour samples, for example, a portion of each of the ten protein lysates is used to create a pooled reference (the 11th sample). To enable comparison across the entire sample cohort, the 11th sample is used in each labelling experiment. Quantitation is achieved by comparing the TMT reporter ion intensities ratios (sample/reference) in each sample.

To increase the analytical dynamic range, proteome coverage and improve quantitation, it is highly advisable to fractionate the peptide mixture prior to LC-MS/MS analysis (Manadas et al. 2010). Currently, the two-dimensional reversed-phase liquid chromatography (2D-RPLC) strategy is the favoured trend in proteomic studies. RPLC exhibits higher peak capacities and resolves peptides more efficiently than other chromatographic systems. 2D-RPLC consists of an initial, first dimension separation with a mass spectrometry–compatible high pH solvent system followed by a second dimension separation with a low pH solvent prior to analysis by LC-MS/MS. This 2D RPLC strategy was applied to our MM proteomic workflow to analyse the TMT-labelled peptides.

As an example, proteins from 10 frozen, sectioned MM tumours were extracted in 100 mM ammonium bicarbonate containing 4 M urea on the Bioruptor. Ten aliquots of the lysate and one from a reference sample pool prepared in advance were placed in the Bravo AssayMAP robot for protein denaturation, digestion and peptide desalting. Protein reduction and alkylation were performed with 10 mM DTT and 20 mM iodoacetamide, respectively. Denatured proteins were digested with endoproteinase Lys-C for 5 h at room temperature using an enzyme:protein ratio of 1:50 (*w*/*w*). This was followed by an overnight digestion with trypsin at room temperature using an enzyme:protein ratio of 1:50 (*w*/*w*). MM samples (each 30 μg peptides) were labelled with TMT 11-plex reagents, mixed and fractionated by high pH RP-HPLC. Eluted peptides were pooled into 24 concatenated fractions. Approximately 1 μg of labelled peptides was analysed by LC-MS/MS on a Q Exactive HF-X mass spectrometer.

The complete LC-MS/MS analysis of the 10 samples including data output was achieved in 3 to 4 days on a single LC-MS/MS instrument. Currently, we can systematically and confidently identify and quantitate > 10,000 proteins. To the best of our knowledge, this represents the largest data set of proteins identified to date from MM tumours.

#### Label-free quantitative proteomics

With the high reproducibility, sensitivity, speed and accuracy of current LC-MS/MS systems based on the orbitrap technology, e.g. Q Exactive HF-X, it is now possible to identify more than 60,000 peptides corresponding to more than 6000 proteins in a single LCMS analysis. These features provide a solid foundation for achieving the highest quality data possible from the quantitative proteomic strategy termed ‘label-free’. To date, the label-free approach is the most straightforward approach for performing quantitative proteomics. There are different label-free quantitation methods available; however, within this article, emphasis is primarily placed on the intensity-based approaches. Intensity-based quantitation is built on the fact that for a given sample, protein abundance correlates with the intensity of the unique peptides (Chelius and Bondarenko 2002). Data acquisition by mass spectrometry was evaluated with data-dependent acquisition (DDA) and data-independent acquisition (DIA).

DDA has largely been the method of choice for high-throughput proteomic analyses. In this method, the mass spectrometer firstly performs a short MS^1^ survey scan of the peptides that are currently eluting from the LC system. This scan monitors peptide ion intensity and identifies potential peptides to be fragmented. A series of tandem mass spectrometry (MS^2^ or MS/MS) events are performed whereby a peptide signal is isolated and fragmented, and the product ions are detected. The peptide intensity and the associated MS/MS data provide the necessary information to identify and quantitate the protein, respectively. Due to the semi-stochastic nature of the ion selection process, several replicates are usually required to increase coverage of the proteome. If a peptide signal is not selected for fragmentation, no MS/MS spectrum is recorded and subsequently these peptide species are not identified. One method to increase peptide selection is to pre-fractionate the sample. Thus, peptide mixtures with reduced complexity are injected onto the LC-MS/MS system. Sample fractionation prior to LC-MS/MS analysis has substantially contributed to increasing the coverage of the proteome (see TMT 11-plex section below).

Recently, DIA methods such as SWATH or MS^e^ have gained increasing popularity. Here, single peptide ions are not isolated; rather, a *m*/*z* window is utilised. The window allows simultaneous fragmentation of all peptides eluting in the selected *m*/*z* range. All product ions from multiple peptide ions are then recorded in a single MS/MS spectrum. To cover a wider *m*/*z* range, several *m*/*z* windows are usually chosen. The result is the generation of highly complex tandem mass spectra. These are compared to previously generated DDA spectral libraries and matched MS/MS spectra are then assigned to peptide sequences.

**Fig. 7:**
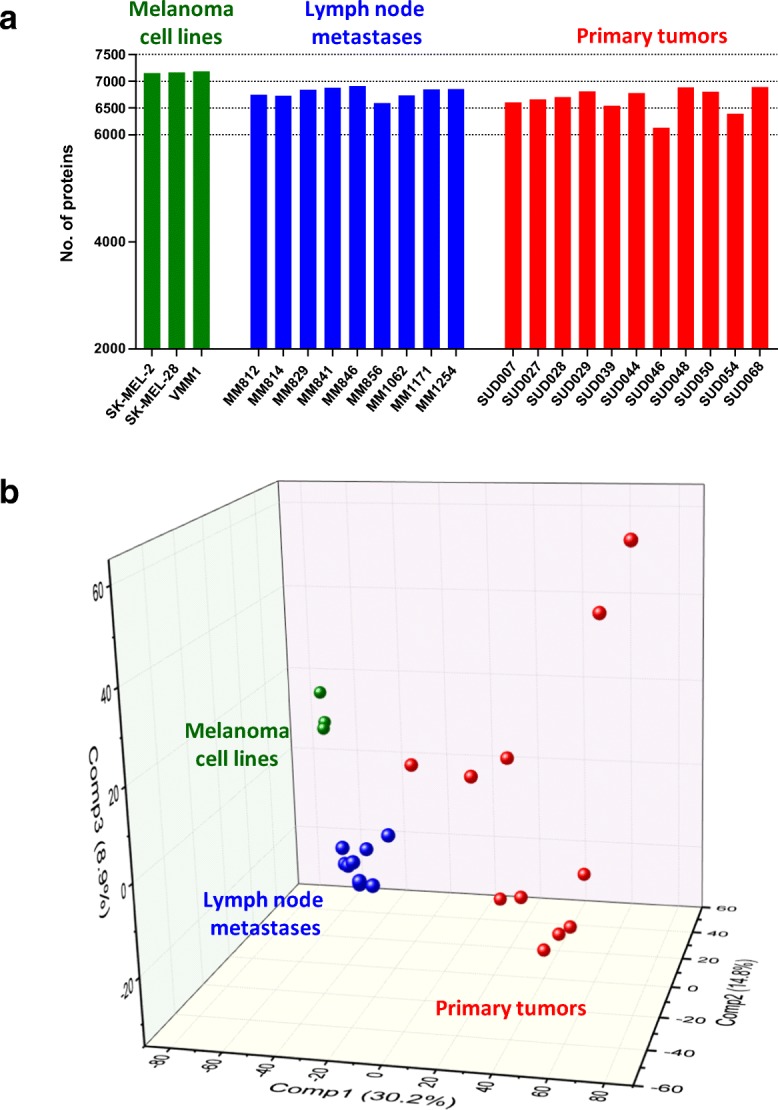
**a** Identified proteins from melanoma cell lines (3), lymph node metastases (9) and primary tumours (11) from an unfractionated total protein digest. Each sample was analysed as a single LC-MS/MS run. The MS data was obtained using a data-dependent acquisition method. **b** Principal component analysis performed with the normalised and standardised abundance intensities of the proteins identified in all samples

A MM cohort including 11 primary tumours, nine lymph node metastases and three cell lines was submitted to a quantitative proteomics analysis based on the label-free approach. The proteins were extracted in the presence of SDS/DTT and after buffer exchange were digested with trypsin. MS data was acquired in a DDA method. The number of identified proteins ranged from 6000 to 7000 in tumour samples, whilst for the cell lines the numbers reached 7200 identifications (Fig. 7a). The relative abundance profiles of identified proteins in all samples were used to create a principal component analysis (Fig. 7b). The results showed that the cell lines and the lymph node metastases cluster together: whilst the primary tumours are more dispersed. The high variability in protein expression observed in primary tumours can provide an explanation as to how and why MM have such a broad range of outcomes. Our data do suggest that at least for the transition from the primary tumour to lymph node metastases, this diversity is reduced. An explanation for this observation may be that not all tumour cells can metastasise or produce a viable metastasis. Thus, the abundance profiles of the proteins in each sample combined with histological data and the clinical and pathological history of the patients will become a powerful tool in understanding the progression of the disease.

### Understanding melanoma by mapping proteomic data on biological pathways and interaction networks

Typically, a proteomic experiment provides a large number of protein measurements that relate to a biological outcome, e.g. exhibit significantly different expression between primary and metastatic tumour. In order to gain insight into the biological meaning of such protein lists, a typical bioinformatic approach involves elucidating over-represented pathways and other functional annotations (e.g. Gene Ontology terms or structural domains).

**Fig. 8:**
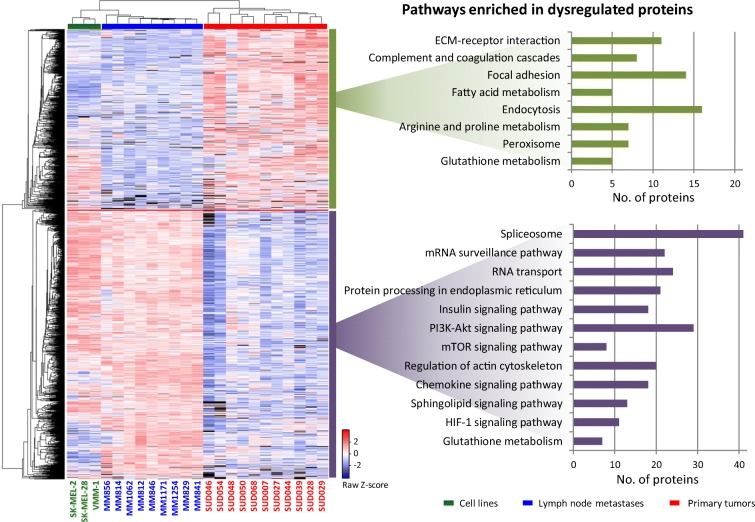
Heat map of the relative abundance of proteins found dysregulated between melanoma primary tumours and lymph node metastases (left). Biological pathways over-represented in proteins dysregulated between the different sample types

The quantitative protein data that was obtained from analysing the MM cohort consisting of 11 primary tumours, nine lymph node metastases and the three cell lines (Fig. 7) contained a wealth of information to aid in understanding the biology and progression of MM. When the samples were grouped according to origin (primary tumours, cell lines and lymph node metastases), more than 1500 different proteins were found to be dysregulated between the groups. In particular, the lymph node metastases had the largest set of upregulated proteins when compared to the primary melanoma tumours. This is presented in the heat map in Fig. 8. Amongst the significantly upregulated proteins in lymph node metastases, pathways such as the spliceosome, RNA transport and mRNA surveillance, i.e. indicative of a higher rate of cell division, were enriched. Most of these proteins were also upregulated in cultured melanoma cell lines. The roles of signalling pathways such as the PI3K-AKT, mTOR and MAPK have been previously described in melanoma (Rodríguez-Cerdeira et al. 2018). In several studies, these pathways were activated in melanoma and other type of cancers. When compared to primary tumours, elements of these pathways showed significant upregulation in lymph node metastases. In this sense, there is a possibility that the upregulation of these pathways could be a prerequisite for the progression of the disease towards metastasis. These results might support the hypothesis that in the primary tumour, only those cells that upregulate these pathways are able to metastasise. These findings partially support the differences in patient survival when the disease is diagnosed at different stages, particularly, if the upregulation of these pathways is only evident in metastatic melanoma.

Proteins expressed in the primary tumours that were downregulated when the disease underwent metastasis (at least to the lymph nodes) were involved in pathways related to cell communication and interaction with the extracellular matrix. In addition, a large number of proteins that are involved in metabolic pathways were over-represented in the primary tumours. The upregulation of peroxisomal and fatty acid metabolism proteins suggested an imbalance in energy production that begins in primary tumours. More profound changes in the metabolism of tumour cells were observed in the metastatic samples. Eleven proteins involved in the hypoxia inducible factor-1 signalling pathway were upregulated in the lymph node metastases. Even in the presence of normal oxygen levels, the activation of this pathway contributes to the metabolic shift from oxidative phosphorylation to the glycolytic phenotype. Amongst the proteins induced by HIF-1, hexokinase 3 (HK3, but not HK1 or HK2) was upregulated in the lymph node metastases and cell lines. HK3 is involved in the first step of glycolysis and is the only isoform not linked to the mitochondria. This means that upregulation of HK3 contributes to the glycolytic phenotype independently to the mitochondrial status. In addition, the phosphoenolpyruvate carboxykinase (GTP), mitochondrial (PCK2) was upregulated in the metastatic samples compared to the primary tumours. This enzyme is involved in the first step of gluconeogenesis and upregulation contributes to the accumulation of glycolysis intermediates that are required to support rapid cell proliferation. When compared to premalignant lesions, similar results in gastric adenocarcinoma biopsies have been found (Fernández-Coto et al. 2018). The activation of glycolysis is also aided by the upregulation of proteins involved in the insulin signalling pathway. When combined with data from more specific disciplines such as phosphoproteomics and acetylomics, the protein expression profiles of samples from different stages of melanoma provide a solid basis for understanding the biology and progression of this disease.

**Fig. 9:**
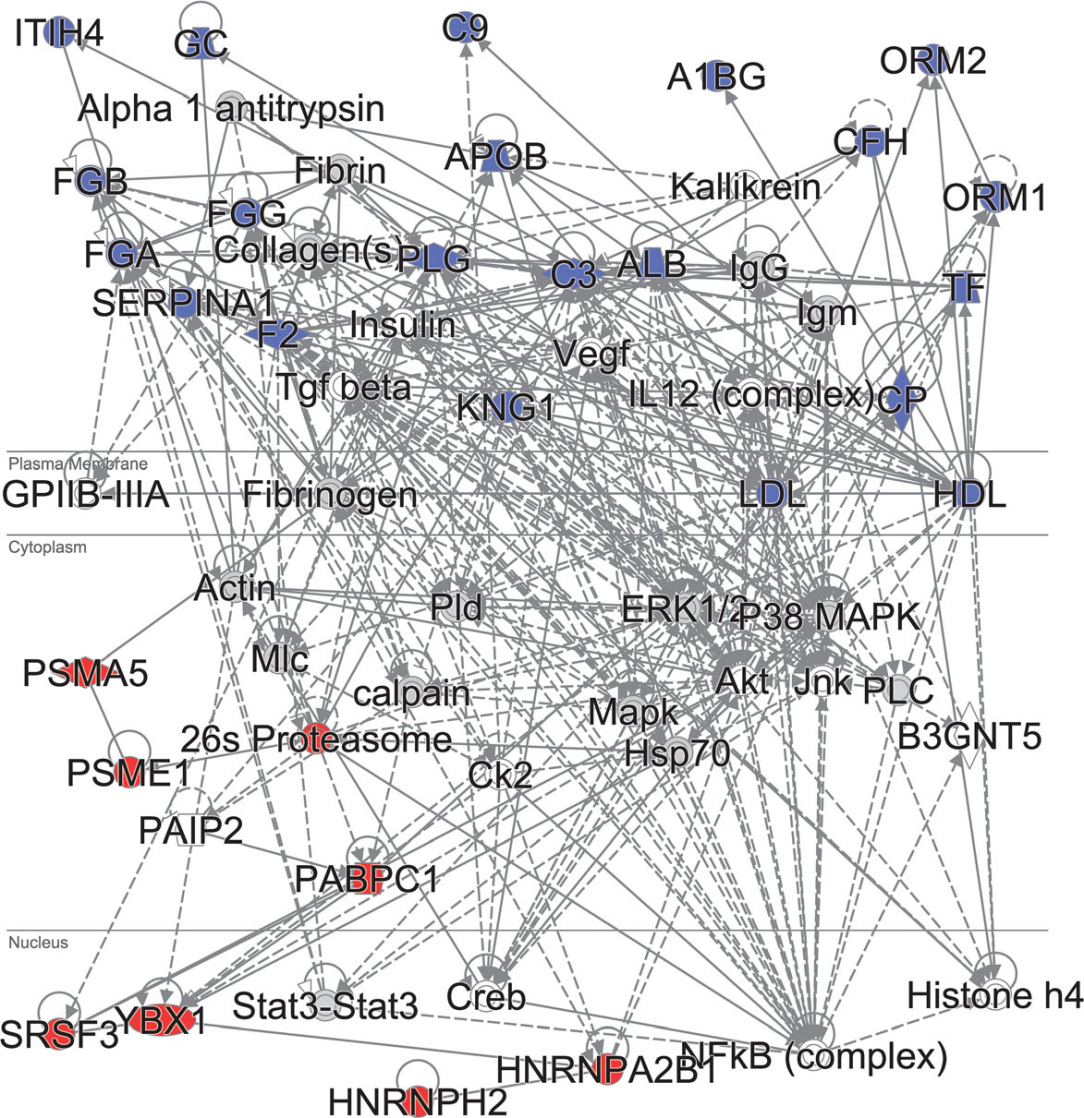
Ingenuity Pathway Analysis (IPA) for the proteins identified by the PLS-Cox analysis as significantly related to survival in a cohort of 111 lymph node melanoma metastases. Two of the top protein–protein relationship subnetworks that are enriched in the query proteins were merged. Blue, proteins with expression negatively correlated with survival. Red, proteins positively correlated with survival. Solid lines, direct relationships. Dashed lines, indirect relationships. Subcellular localisation is indicated

For a more unbiased view, the proteomic data can also be mapped on biological relationship networks that may include protein–protein interactions, activation, post-translational processing or influencing expression. Such relationship networks can be built by literature curation, or automatically by integrating data from various databases. As an example, in Fig. 9, such an analysis is presented for a set of proteins that had an expression pattern that was significantly related to patient survival in a cohort of 111 lymph node metastasis samples from patients with different melanoma survival histories (*Betancourt* et al., *manuscript under review*). Here, a partial least squares–Cox regression (PLS-Cox) model was built that reduced the expression of an entire feature set (~ 1300 proteins) to a single inferred variable. This subsequently explained the main reason for protein expression variability with respect to patient survival. The survival-related proteins were used as queries for a large functional relationship database (Ingenuity Knowledge Base). Querying (Ingenuity Pathway Analysis) involved extracting dense relationship subnetworks enriched in the query proteins. Amongst proteins that positively correlated with survival (high expression in longer surviving patients), mapping to relationship networks identified small groups of transcription factor, splicing factors and proteasome subunits that most probably regulate tumour development and can be promising biomarkers. Proteins negatively correlated with survival (high expression in shorter survival) are primarily functionally related extracellular proteins with expression that may be linked to the vascularisation aspect of melanoma metastases and to immune component of cancer.

The network mapping approach not only provides functional modules composed of subsets of query proteins that are likely to act together in the biological process studied but also merges these with non-query proteins that are nevertheless tightly functionally interconnected with the queries.

### Post-translational modification of proteins

#### Pathway signalling and protein phosphorylation

Regulation of molecular events and protein dynamics are commonly associated with PTMs (Ardito et al. 2017). From the ~ 200 known PTMs, phosphorylation is one of the most studied and documented (Sharma et al. 2014). Phosphorylation involves the addition of a phosphate group onto the side chain of serine, threonine and/or tyrosine residues (Ubersax and Ferrell 2007). This modification is usually mediated by the action of kinases and phosphatases (Hunter 1995), and is involved in multiple biological functions including migration, cell growth, differentiation and cell death (Ardito et al. 2017). These functions are usually performed by the action of several signalling pathways (Tarrant and Cole 2009).

**Fig. 10:**
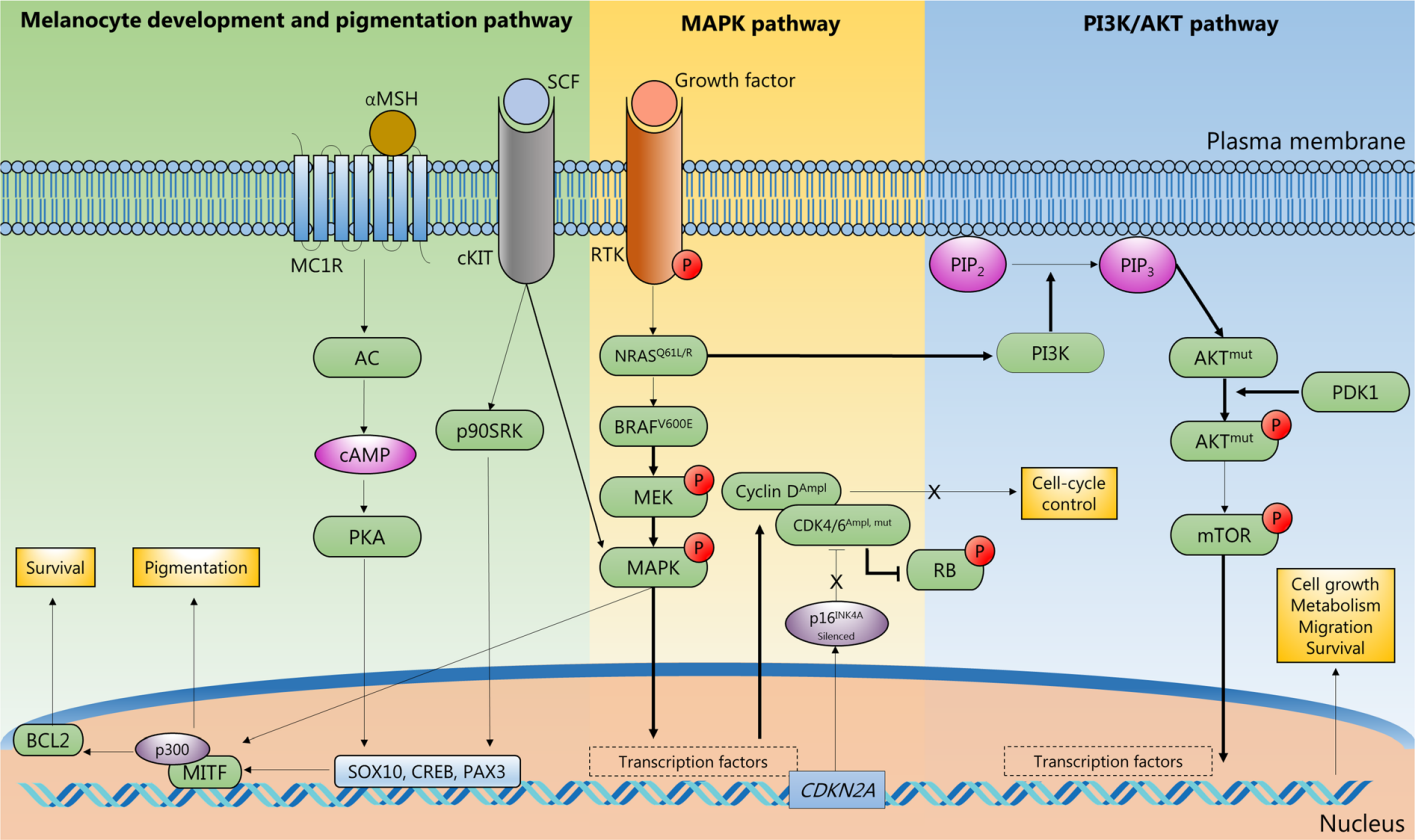
Illustration of pathway signalling where phosphorylation signalling PTMs have been sequenced and annotated in melanoma tumours from patients

MM induces abnormal activation of signalling pathways that affect the overall phosphorylation profile of the cells (Rodríguez-Cerdeira et al. 2018). In this context, the application of phosphoproteomics to MM has become extremely relevant. In order to block these pathways, protein targets against which new drugs can be designed must be identified (Abelin et al. 2016). The RAS/RAF/MAPK (mitogen-activated protein kinases) pathway appears to be a key regulator of the development of MM. MAPK proteins are essential in cell proliferation and evasion of apoptosis (Burotto et al. 2014). The classical MAPK pathway includes proteins such as v-Raf murine sarcoma viral oncogene product (BRAF) and the downstream partners extracellular signal-regulated kinases 1 and 2 (ERK1/ERK2) (Burotto et al. 2014). These proteins activate several transcription factors involved in cell development and proliferation (Fig. 10). BRAF has received enormous attention because of the mutation rate in MM patients (50–60%) (Hu-Lieskovan et al. 2014). In addition, some drug therapies based on BRAF inhibition or combined BRAF and MEK inhibition against MM have been successfully applied in the treatment of melanoma (e.g. vemurafenib, trametinib, dabrafenib and vemurafenib with cotellic) (Chapman et al. 2011; Hauschild et al. 2012; Hu-Lieskovan et al. 2014).

Knowledge on the MM phosphoproteome was generated by applying phosphopeptide enrichment protocols and LC-MS/MS on MM-derived cell lines (Basken et al. 2018; Galan et al. 2014; Smit et al. 2014). Amongst the methodologies available to enrich phosphopeptides in MM, the most widely practiced are immobilised metal ion chromatography (IMAC) (Thingholm and Larsen 2016a) and titanium dioxide (Thingholm and Larsen 2016b), and combinations thereof. In addition, fractionation protocols such as strong cation exchange (SCX) (Lombardi et al. 2015), hydrophilic interaction liquid chromatography (HILIC) (Boersema et al. 2008) or basic reversed-phase chromatography can be utilised to increase coverage of the phosphoproteome (Batth et al. 2014). To perform phosphoproteomics, large quantities of protein are usually necessary (Mertins et al. 2013). Consequently, cultured cells are usually preferred. To characterise the MM phosphoproteome in detail, and to discover biomarkers of clinical significance, large MM patient tissue cohorts are required. Unlike cell lines, patient tissue takes into account the heterogeneity of the tumours and the microenvironment of the surrounding cells (Marcell Szasz et al. 2018; Szász et al. 2017). This, however, raises additional challenges such as reproducibility, low quantities of available starting material, material loss and lack of automation.

To overcome these challenges, a protocol to perform phosphopeptide enrichment from MM tissues in an automatic manner was recently optimised (Post et al. 2017). The AssayMap Bravo platform enables enrichment of phosphopeptides with high selectivity and sensitivity, and up to 96 samples can be simultaneously processed (De Graaf et al. 2016; Post et al. 2017). From 16-fold less material than previous reports on cell lines, thousands of phosphopeptides were detected using Fe(III)-IMAC cartridges. The obtained phosphoproteome covered essential features of MM (MAPK and non-canonical MAPK phosphoproteins) and was comparable with previous reports based on cell lines. More importantly, additional MM-related pathways were revealed that were only apparent in human tissue (i.e. anti-tumour immune response). The development and incorporation of this protocol represents a significant step towards a better understanding of MM and an opportunity to integrate clinical data combined with phosphoproteomics in biomarker screening.

#### Quantitative acetylomics

Protein lysine acetylation is a widely spread PTM that participates in the regulation of a vast majority of cellular processes. Lysine acetylation is a reversible PTM that occurs on the epsilon (ε) amino group of lysine residues. This PTM is catalysed by lysine acetyltransferases or via non-enzymatic mechanisms under a specific chemical environment, e.g. the mitochondrial matrix. The removal of an acetyl group only occurs enzymatically. Two groups of enzymes with lysine deacetylase activity have been described: the Zn^2+^-dependent lysine deacetylases and the sirtuins. The latter uses NAD^+^ as a cofactor. Lysine acetylation affects the interaction of a modified protein with other molecules such as nucleic acids, or by opposing the occurrence of other PTMs targeting the same site, e.g. methylation and ubiquitination.

There is an increasing number of studies linking dysregulation of lysine acetylation targets, interacting proteins thereof and controlling enzymes with the development and progression of cancer. Particularly in melanoma, class I lysine deacetylases are overexpressed when compared to non-cancerous cells (Krumm et al. 2016). Greater efforts, however, are imperative to fully understand how the upregulation of these enzymes alters the acetylation status of the targets and how this is subsequently translated into cancer progression. Therefore, the study of not only the expression of these enzymes but also targets thereof and the status of the acetylation modification will open new avenues in terms of therapeutic strategies.

**Fig. 11:**
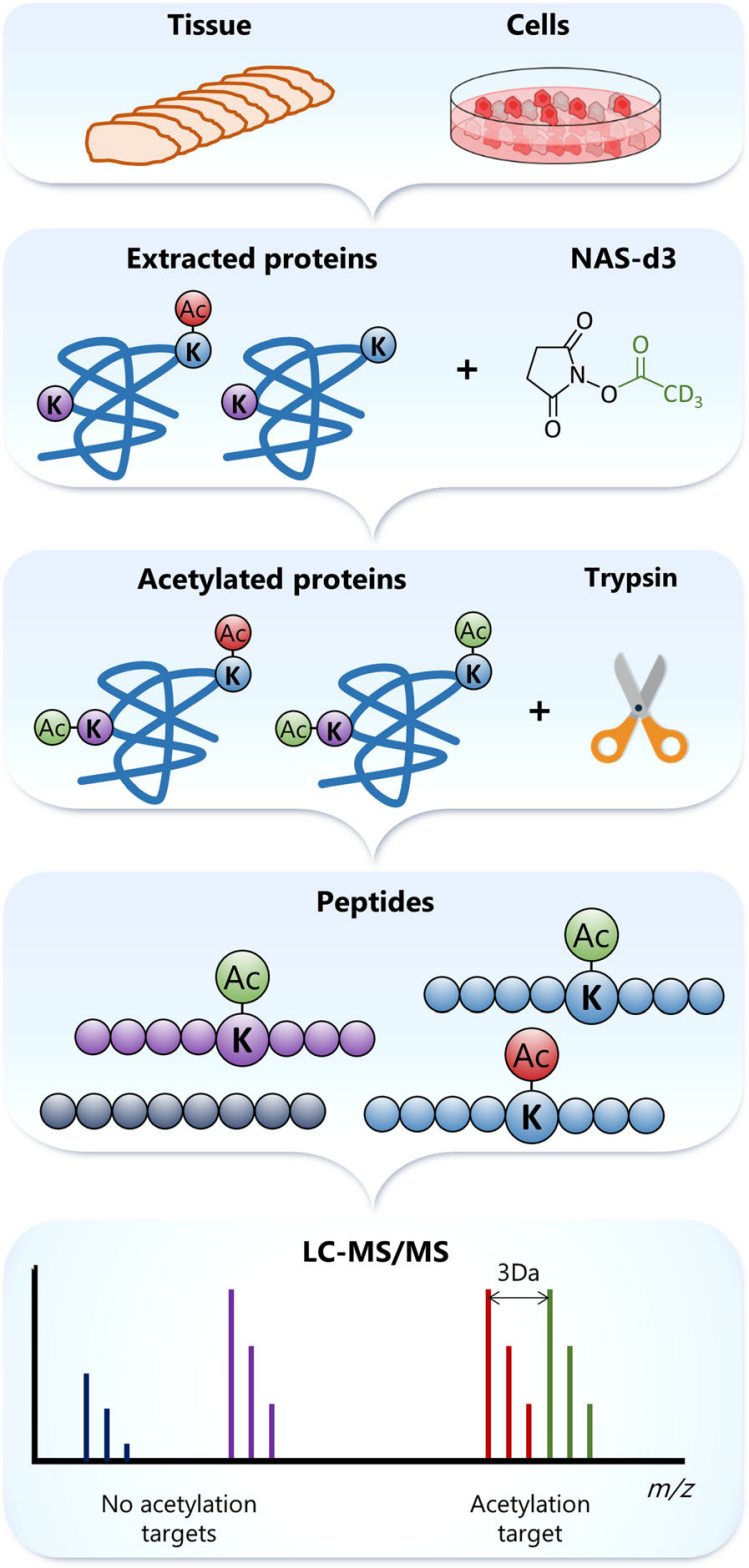
Strategy to identify and determine the stoichiometry of acetylation sites

To study lysine acetylation, an MS-based approach aimed at identifying and quantitating lysine residue acetylation was implemented (Gil et al. 2017). This method relies on the chemical modification of the free ε-NH_2_ groups in proteins by acetyl groups labelled with deuterium (CD_3_–CO–) (Fig. 11), and subsequent differentiation from endogenous acetylation (CH_3_–CO–, Fig. 11). The acetylation of all lysine ε-NH_2_ groups in proteins limits trypsin cleavage to arginine residues. The complexity of the sample is subsequently decreased by reducing the number of generated peptides per protein. With a reduced number of diverse peptides per protein in the sample, the LC-MS/MS system can, in theory, analyse peptides from more proteins, which additionally aids in the identification of lower-abundance proteins. After LC-MS/MS analysis of the tryptic digest, the identification of mass signals assigned to every peptide containing deuterium-labelled acetylated lysine triggers the search for peptide signals at the same elution time with a decrease in mass of 3 Da, i.e. the mass difference between the endogenously and exogenously labelled acetylated peptides. A thorough informatic analysis of the mass accuracy and isotopic distribution of the signals enabled both the identification of the acetylated lysine residue and the extent of the modification.

Using this approach, the acetylation sites and the stoichiometry of the modification were determined for protein lysates from different melanoma cell lines. More than 2200 acetylated peptides corresponding to more than 1500 different proteins were identified in each cell line. In addition, our results confirmed previous findings that lysine acetylation is a PTM with low stoichiometry (Schölz et al. 2015). More than 40% of all identified acetylation sites showed less than 10% occupancy by this PTM. Amongst the acetylated proteins were found those directly linked to ribosomal RNA processing. Others are involved in the spliceosome machinery and mRNA splicing. Ribosomal proteins were also confirmed as group influenced by acetylation.

### Liquid biopsies in melanoma

Liquid biopsies refer to the analysis in blood or other body fluids, of molecules or cells as a result of tumour leakage (Schwarzenbach et al. 2011). Nucleic acids such as DNA, mRNA and microRNA can be detected circulating in blood and they can be released from cancerous cells. However, not only tumour cells, but also other pathological and physiological conditions, can result in an increase of these molecules in blood. In this sense, in melanoma, the analysis of circulating DNA from tumour has been focused mainly on the detection of driver gene mutations, e.g. BRAF and NRAS (Buder-Bakhaya et al. 2017). This type of study has potential to be used for prognosis (Gray et al. 2015). In several studies monitoring mutated BRAF DNA in plasma from melanoma patients, correlation has been found between levels of circulating DNA and response to treatment with BRAF inhibitors, as reviewed by Calapre et al. (2017). The detection of other nucleic acid molecules in melanoma liquid biopsies has been less explored. Even though liquid biopsies have intrinsically prognostic value, the potential usage for monitoring disease progression as well as predicting different outcomes, liquid biopsies have still not yet been established as a routine clinical test.

## Comprehensive data processing and analysis

**Fig. 12:**
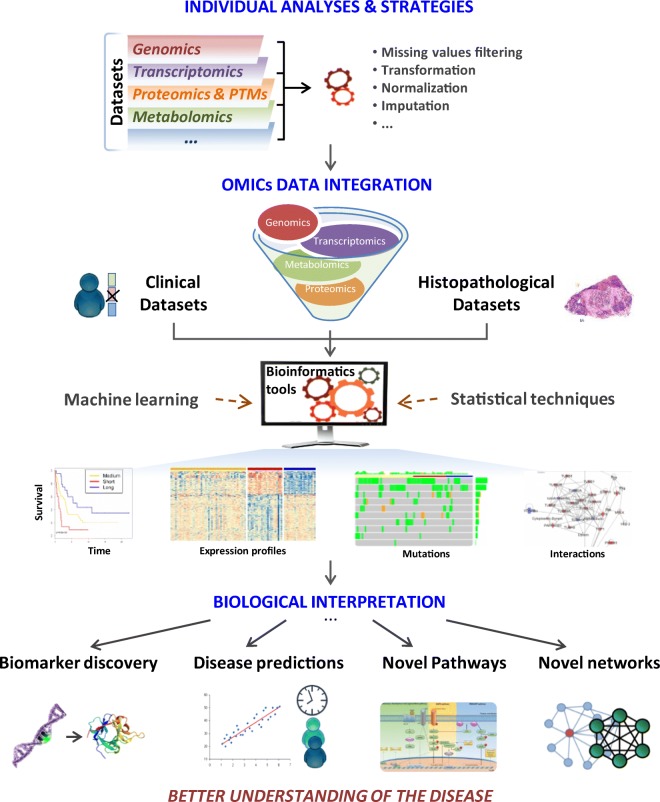
Data integration and most common outcomes. Omic and clinical information combined with histopathological data are integrated through bioinformatic tools based on machine learning and statistical approaches. As a result, it is possible to discover new biomarkers and to obtain a better understanding of the disease

Cancer research projects can include several ‘omic’ strategies such as proteogenomics, transcriptomics and metabolomics. Together with clinical and histopathological data, the information from these platforms has broadened our knowledge about the entire biological systems. The most efficient, albeit challenging, way to study such systems is to integrate different data sets that were generated from several complementary techniques (Fig. 12).

Currently available tools to integrate omic data include web-based tools requiring no computational experience, and more versatile tools for those with computational experience (Misra et al. 2018). Examples of such tools include the more user-friendly Paintomics (http://www.paintomics.org), 3Omics (http://3omics.cmdm.tw/) and GalaxyP,M (https://usegalaxy.org/), and IntegrOmics (http://math.univ-toulouse.fr/biostat), SteinerNet (https://cran.rproject.org/src/contrib/Archive/SteinerNet/), OmicsIntegrator (http://fraenkel.mit.edu/omicsintegrator,https://github.com/fraenkel-lab/OmicsIntegrator) and MixOmics (http://mixomics.org/) for users with programming expertise.

In this melanoma project, the patient samples were analysed with several analytical techniques, each with different aims and approaches. By applying machine-learning tools and statistical approaches, correlations between variables from the different data sets (e.g. proteins panel and % of tumour) were determined. Ultimately, this type of information leads to the discovery of new biomarkers.

### Proteogenomics

Proteins are the downstream products of the genome and transcriptome. Protein properties such as the primary sequence can be predicted (with high accuracy) from the genome and proteome. Properties such as post-translation modifications or protein quantity in a particular cell location or tissue are the result of complex interactions with other proteins, environmental effects and stimuli and therefore cannot be accurately predicted. The chemical composition of proteins is more complex than the genome, and consequently, proteins are more challenging to qualitatively and quantitatively analyse. Proteins, however, are the molecules that actively contribute to biological events. Therefore, these harbour important information to further our understanding on developmental, ageing and disease processes in living organisms.

The genome, transcriptome and proteome do share similar information on the state of a living organism, but additional different types of complementary information can also be determined (Zhu et al. 2018). The goal of proteogenomics is to integrate tightly qualitative (identification) and quantitative aspects obtained from genomic, transcriptomic and proteomic information.

#### Tracking disease-associated mutations and/or single amino acid variants in malignant melanoma

Qualitative aspects encompass identification of sequence variants that include, e.g. single amino acid polymorphism, splice junction peptides and rare sequence variants. These variants cannot be identified with a standard proteomic workflow because canonical sequences from public databases such as Uniprot or Ensembl are used. The canonical sequence is the longest protein sequence and is usually the most abundant form of a protein-coding gene (SwissProt) or contains a limited number of proteoforms (Trembl). Identification of these variants can be performed in different ways. One of the most popular approaches is to complement the canonical sequence with sequence variants from various databases such as CanProVar1.2 (Li et al. 2010; Zhang et al. 2017), VarCards3 (Li et al. 2018), COSMIC4 (Forbes et al. 2017) and others (Yang et al. 2015). Alternatively, 6-frame translation of the human genome or 3-frame translation of mRNA data from various samples can be included (Kim et al. 2014; Nesvizhskii 2014; Zhu et al. 2018). Other approaches use genomic or transcriptomic data obtained from the same sample to predict protein sequence variants. The latter approach is preferred as a smaller search space is utilised (including only non-synonymous variants present in the sample) and the statistical scoring of the database search is facilitated (Low et al. 2013).

Previous studies have estimated that the mutation rate for melanoma and lung cancer cell is up to 100 per 1 million base pairs. Thus, a mutation phenotypically expressed at the protein level is invaluable as this may potentially represent a novel drug target. The problem of drug resistance (*a.k.a.*, why do some patients respond to a particular treatment whilst others do not?) represents another remarkable question that can be addressed through proteogenomic studies. For example, MM is characterised by somatic BRAF and RAS mutations in the MAPK pathway. These mutations strongly correlate with poor prognosis of the disease. The inhibition of the mutated BRAF with selective inhibitors such as vemurafenib or dabrafenib has resulted in the reduction of MAPK signalling and regression of the disease. Unfortunately, most patients quickly develop resistance to drug treatment and the identification of proteins with somatic mutations that influence the development of resistance has largely remained elusive (Salemi et al. 2018).

In MM translational research to date, only a few reports have utilised a proteogenomic approach. In a pioneering study, Lobas et al. (2018) used MM cell lines to identify protein variants originating from coding mutations. This was achieved by acquiring and processing exomic and deep proteomic data. The authors also assessed strategies to minimise both false-positive and false-negative identifications, an important goal in cancer proteogenomics.

**Fig. 13:**
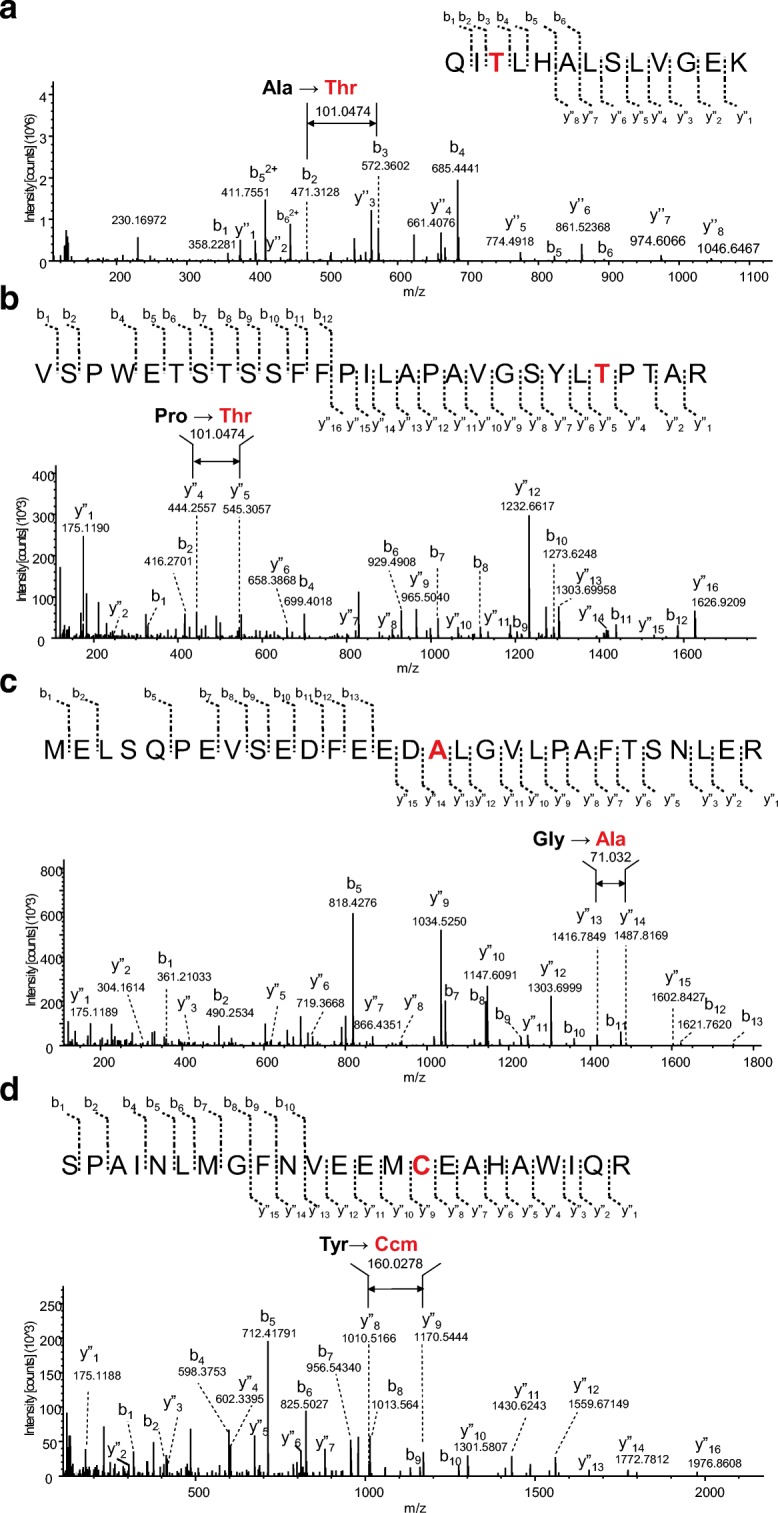
MS/MS spectra of peptides from subunits 4 and 9 of poly ADP-ribose polymerase (PARP) confirming the occurrence of mutations and single amino acid substitutions in the sequences. **a** Substitution of the alanine residue at position 899 for a threonine in PARP-4, **b** substitution of a proline residue at position 1328 for a threonine in PARP-4, **c** substitution of a glycine residue at position 1265 for an alanine in PARP-4, and **d** substitution of a tyrosine residue at position 493 for a cysteine in PARP-9. The designation for the fragment ion signals in the MS/MS spectra is according to the Roepstorff–Fohlmann–Biemann nomenclature

Preliminary searches for protein isoforms and mutant variants were performed on the proteomic data generated from regional lymph node metastatic melanomas. Single amino acid variations were observed in a significant number of proteins. In Fig. 13 are the observed protein variants of the poly ADP-ribose polymerases (the PARP protein family) that were identified in at least 20 of the analysed metastatic melanoma tumours. PARPs play a role in chromatin modification, DNA replication and transcription during induced cell death and DNA repair. The most prevalent isoforms of the poly ADP-ribose polymerase were detected in our proteomic study, a fact that indicated the complexity and heterogeneity of the disease at the protein level.

Given the capacity to target BRCA1/2-deficient cancers, PARP inhibitors (PARPis) have elicited considerable enthusiasm as a cancer treatment, and the discovery of PARPis led to the concept of ‘synthetic lethality’ (Helleday 2011). In fact, inhibitors of the PARP family of proteins are currently under preclinical and clinical evaluation as anticancer medication for melanoma and ovarian, breast and prostate cancer. PARPis have a peculiarity in that these drugs increase the efficacy of DNA-damaging agents to selectively target tumour cells with specific DNA repair defects (Musella et al. 2018; Papeo et al. 2013; Plummer et al. 2013). Resistance of cancer cells to PARP inhibitors, however, is also beginning to occur and accurate biomarkers for treatment sensitivity and resistance remain challenging (Montoni et al. 2013; Schlacher 2017).

#### Linking genes to protein expression and function

Quantitative aspects include co-expression (correlation) analysis between the two layers, which is known to be medium (around 0.4–0.5 in cell lines and tissue). This medium correlation can be explained by the fact that transcripts are upstream to proteins and transcriptions, translations and post-translation and protein and transcript degradation are events that have different time dynamics (Schwanhäusser et al. 2011).

Other integration approaches use multivariate statistics that aim at performing dimension reduction to integrate heterogeneous quantitative molecular profiles and use the obtained model for prediction of, e.g. survival or treatment efficiency, or perform improved disease classification. Examples of such methods are Multiview Nonnegative Matrix Factorization Algorithm (Ray et al. 2017), Joint Non-negative Matrix Factorization (Zhang et al. 2012; Zhang et al. 2011) and mixOmics R package (Rohart et al. 2017). Compound co-expression networks based on correlation or partial correlation (Graphical Gaussian models, GGM) are also gaining momentum. The NetICS method can be used to identify, e.g. mediator genes, and translate upstream events, e.g. differential expression, genetic mutations and differential methylation of the gene promoter region (Dimitrakopoulos et al. 2018). The reader is invited to read recent reviews on the various statistical approaches in Bersanelli et al. (2016) and Huang et al. (2017).

Other aspects of proteogenomic data integration are to reveal the effect of genomic variants on transcript (eQTL) and protein (pQTL) abundance on the basis of quantitative trait loci analysis and the effect of gene duplications/deletions for both molecular layers using copy number variation (CNV) analysis (Mertins et al. 2016). These analyses have the potential to reveal somatic or germline variants and chromosomal aberrations that alter transcript and protein abundance and link these to the disease.

Another interesting direction of linking genes to protein expression and function is the analysis of DNA methylation. DNA methylation is an epigenetic mechanism that occurs mostly by the addition of a methyl group to DNA at the 5-carbon of the cytosine ring. There is growing evidence demonstrating that DNA methylation may deeply alter protein expression and potentially be a causative event in cancer (Fernandez et al. 2012; Jones and Baylin 2002). Hypermethylation has been associated to the silencing of genes and to decreased gene expression of tumour suppressors, whilst hypomethylation can potentially result in genomic instability and reactivation of oncogenes (Litovkin et al. 2014, 2015; Paska and Hudler 2015; Weisenberger 2014). Based on genome-wide studies, abnormal methylation patterns have been detected in melanoma patients, highlighting potential markers for disease progression and also providing an important strategy for tumour diagnosis and treatment (Fu et al. 2017; Guo et al. 2018; Koga et al. 2009). Nevertheless, as the proteome represents a link between DNA and phenotype, proteins likely provide a more accurate depiction of the cell state. Hence, integrating next-generation sequencing applications in methylome analysis with comprehensive proteomic data provides an opportunity to more accurately identify functionally relevant abnormal methylation events that drive cancer pathogenesis.

## Challenges in drug discovery and development

The Food and Drug Administration (FDA) and other international agencies are posing ever-increasing demands prior to approval and release of novel drugs onto the market. Ultimately, these demands improve quality of care and treatment outcome for patients. There are a number of challenges and pitfalls where drugs prove to be unsuitable for medical use in healthcare. Managing these is of mandatory importance to the medical industry, and recently, a report outlined in detail a benchmark survey of a large number of drug development projects in phases I–III. From this, the 5R Framework strategy was implemented (Cook et al. 2014; Vreman et al. 2018). The 5R Framework encompasses the right target, right patient, right tissue, right safety, and right commercial potential. The right protein target refers to the need for evidence of a strong link between a disease and the chosen protein target. In addition, the right level of safety is important to provide both differentiated and clear safety margins. This also encompasses understanding secondary pharmacological risks that may occur from reactive metabolites and genotoxicity (Cook et al. 2018). The right tissue must be identified to enable adequate bioavailability and tissue exposure and where the pharmacokinetics is well understood. The right patients are imperative to identify the most relevant population where the highest drug efficacy can be obtained. The most difficult component of 5R is to address the safety issues associated with novel drugs. This is often the major reason for failure and accounts for at least 50% of all project closures (Arrowsmith and Miller 2013; Cook et al. 2014; Paul et al. 2010). Additionally, safety and lack of efficacy indirectly contribute to project closure by limiting the dose at which compounds can be evaluated in humans. This results in a prevention of adequate drug exposure that also limits protein target engagement.

As an example, epidermal growth factor receptor (EGFR) tyrosine kinase inhibitors (TKIs) such as gefitinib (IRESSA) and erlotinib (TARCEVA) are established treatments for advanced non-small-cell lung cancer (NSCLC) in Asia (Han et al. 2014; Kato et al. 2011; Mok et al. 2009). The EGFR-TKIs exhibit high-affinity binding to the mutated EGFR tyrosine kinase domain and have been used as an approach to treat advanced NSCLC in Japanese populations (Dagogo-Jack et al. 2018; Nogami et al. 2019). Understanding the mode-of-drug action is a key component in safe and effective treatments, and the measurement of biomarkers will become very important. Utilising liquid chromatography–mass spectrometry, a recent study was undertaken to identify plasma biomarkers for interstitial lung disease in NSCLC patient groups (cases and controls). This is likely to be one of the largest biomarker discovery studies performed to date by mass spectrometry (Marko-Varga et al. 2007; Nyberg et al. 2011; Végvári and Marko-Varga 2010). The success of the aforementioned personalised medicine studies has established a new paradigm in these Asian healthcare centres: the right medicine to the right patient at the right time point.

### Drug imaging by mass spectrometry

The development of more effective drugs is at the heart of any future plan to meet societal pressures and necessities. Our research endeavours are positioned to provide healthcare decision-makers with new options with better medicines. Academic research has contributed greatly to the drug development process by identifying potential disease targets and developing the technology platforms that support and validate the eventual clinical products. There are many diverse disciplines that participate in this process, from clinical medicine to molecular biology, and also chemistry, physics, statistical sciences, informatics and device engineering. To provide the overall scientific knowledge required to launch a new pharmaceutical product, these disciplines must be highly inter-connective and mutually co-dependent. The development of new technologies that can aid in selecting effective candidate drugs would provide broad benefit to:

- Improve the success rate for approval by the FDA and regulatory agencies
- Identify safety issues with certain compounds
- Reduce the cost of drug development by identifying problems early in the decision-making process
- Increase our understanding of the mechanisms of drug efficacy

At the very centre of the drug development process is the matching of specific biological targets with an antagonist or agonist drug. This interaction must result in a biological effect that limits or suppresses the pathology of the disease. Nevertheless, many questions arise. How do we know what happens to a drug once administered to patients? What is the fate of the drug? Where does the drug accumulate and what effect is there at the local site of target interaction? What other sites does the drug also affect? For most of the drugs on the market, there is a paucity of information on the actual localisation of administered compounds within the multitude of different types of tissue compartments in humans. This information can be obtained in experimental animal studies; however, extrapolating data from animal models to humans is not without limitations and problems.

Although the last half century has witnessed dramatic advances in the field of medical imaging, there is still an urgent need to develop more advanced techniques to image compounds during the drug discovery process. This is particularly important in order to narrow the selection of potential leads for further development. One of the reasons this has been difficult to accomplish in the past is that the only avenue for visualising the in vivo distribution of drugs in targeted tissues was to use radioactive labels that inherently pose a health and safety risk. The standard methods to localise drugs in situ have included autoradiography and positron emission tomography (PET). These can provide information on the distribution of a radio-labelled compound even at the cellular level; however, both methods rely on quantitative data that is based on the relative strength of the label rather than the relative concentration of the drug. If a drug is metabolised and the label remains on a metabolised fragment of the drug that is not active, not the precursor of an active form, then the read-out of distribution may have little to do with the mode of action or the actual efficacy of the drug. Other methods rely on isotopes with relatively short half-lives or fluorescent tags that hinder long-term pharmacokinetic analyses or alter the chemical structure and thus the binding affinity and/or avidity to the target molecule. From this point of view, it is particularly important that the methods chosen should investigate the characteristics of the unaltered native compound (i.e. the same agent that is administered to patients).

Particularly for drugs, mass spectrometry imaging (MSI) is a technology under rapid development and this particular combination is finding widespread application across the life and medical sciences (Charkoftaki et al. 2018; Gessel et al. 2014; Végvári et al. 2011). Recently, important progress has been achieved in tissue preparation combined with novel MSI instrumentation to generate high-quality data. In addition, MSI can now aid in interrogating the spatial distribution of native drug compounds and drug metabolites within tissues at the resolution of a single cell (Sugihara et al. 2018; Torok et al. 2015, 2017; Végvári et al. 2017). Cold compounds are being measured by MSI, i.e. without any modification of its native structure.

In drug imaging, the signals that are generated and acquired as a mass spectrum will spatially define the distribution of a drug within the tumour tissue. The resultant data reflects the pattern of a specific compound within the tumour compartment and is based on a profile of intensity values that correspond to specific mass-to-charge (*m*/*z*) values. The technology that enables the generation of modern drug imaging spectra is capable of accurately computing high-resolution *m*/*z* data to approximately the mass of a single electron.

By understanding mode-of-drug-action mechanisms, efficacy prediction can be improved. Thus, imaging mass spectrometry can aid in the development of safety strategies for the treatment of melanoma. Together with establishing large biobanks, there will be considerable improvements in patient treatment. A major goal, therefore, is to provide drug mechanism evidence by drug-target affinity interaction and protein target binding specificity and redundancy. These are the key elements of efficacy and safe drug use. Our integrated system combines macroscopic and microscale imaging with drug and mechanistic information that predicts responders.

Over the last years, our research group has become one of the world leaders in developing a new method to identify, quantitate and determine the in vivo tissue distribution of label-free drugs and metabolites by mass spectrometry imaging of histological tissue sections.

**Fig. 14:**
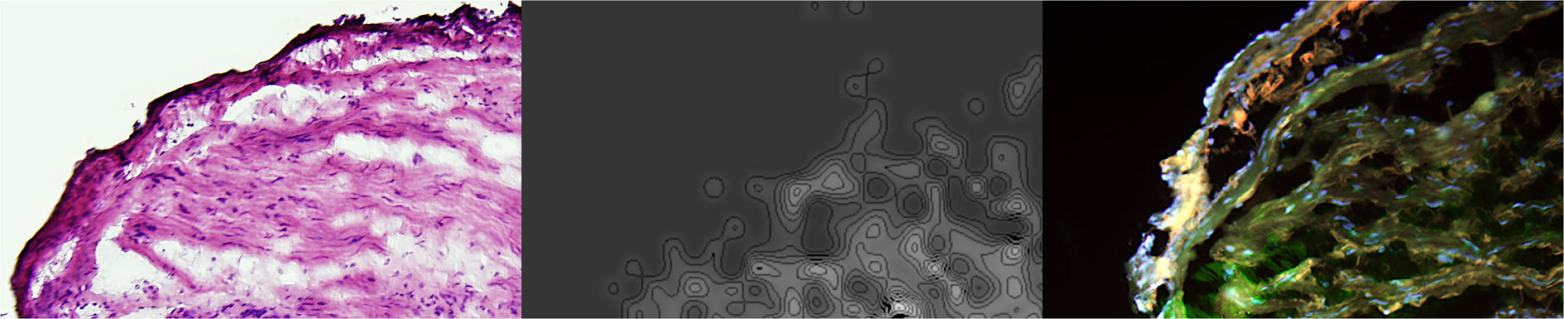
First reported study where MALDI-MSI was used to demonstrate localisation of a drug administered at therapeutic levels in humans. The study demonstrated that the ipratropium precursor ion (*m*/*z* 332.332) is rapidly absorbed into the airway wall partitioned within submucosal spaces containing the targeted airway smooth muscle

Dedicated MSI laboratories have been established within pharma industry, where instrument platforms have been developed to routinely provide detailed spatial quantitation of drugs within tissue via mass spectrometry imaging (MALDI-MSI). MALDI-MS has been used since the mid-1990s in pharmacokinetic studies to characterise in vitro metabolites. As an important advancement in studying drug action, MALDI-MSI can determine the spatial distribution of drugs and metabolites thereof at a histologically relevant resolution. MALDI-MSI does not require labelled compounds and thus can be applied to native drug structures and metabolites within any tissue environment. Tissue biopsies are an important component of the diagnostic evaluation of many conditions and, until recently, analyses performed on tissue samples typically focused on the histology, pathology, gene expression and immunohistochemistry for biomarker expression. The measurement of the drug level in tissue by MSI is a standard approach complemented with the above-mentioned indices. Using this methodology, the first report on tracking an unlabelled drug to the targeted tissue compartment was achieved by our research team (Fehniger et al. 2011). In this study, an inhaled anti-muscarinic receptor antagonist, ipratropium, was tracked to the receptor located on the bronchiole wall smooth muscle bundles. This imaging modality has also been combined with in situ labelling of the target receptor (Fig. 14).

MALDI-MSI is more and more widely used to describing drug distributions in experimental systems. Nevertheless, more than 100 reports have been published where the technology has been used to characterise drug compounds in experimental drug predicting models (Connell et al. 2015; Kwon et al. 2015; Marko-Varga et al. 2012a; Végvári et al. 2017). Until recently, only our own studies have described such drug distribution in humans. The ability to analyse the same tissue section with multiple assays such as MALDI-MSI (drug or marker), immunohistochemistry (protein target, antigen, receptor), conventional pathology chromogenic stains (histology, image analyses) and in situ hybridisation (gene expression, etc.) provides an incredible and rare opportunity to study and define biology at the very local level of histological compartments.

**Fig. 15:**
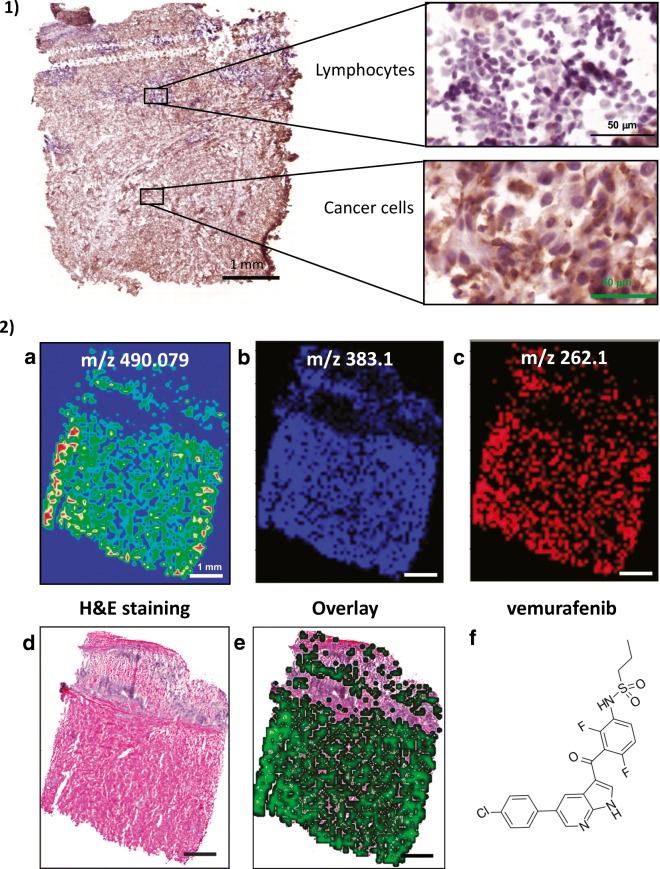
(1) Immunohistochemistry image of a frozen melanoma tissue from a lymph node. BRAF V600E specific antibody was used for immunohistochemistry with DAB stating. BRAF V600E was expressed in the cytoplasm in the melanoma cells. The lymphocytes were used as a negative control. (2) Vemurafenib distribution in melanoma tissue. Adjacent tissue sections were used for immunohistochemistry. (a) High-resolution MSI spectrum of vemurafenib (*m*/*z* 490.079); (b and c) low-resolution ion trap MS/MS data for two fragment ions of vemurafenib (*m*/*z* 383.1 and 262.1); (d) haematoxylin and eosin (H&E) staining demonstrated the distribution of the cancer cells and lymphocytes; (e) overlaid image of the MSI vemurafenib distribution and histology showed the vemurafenib signal originated from the melanoma cells and not the lymphocytes; (f) chemical structure of vemurafenib

Drug selectivity and specificity is dependent on the frequency of a protein mutation and the specific binding properties of the drug molecule. The ultimate proof of personalised medicine (PM) mechanisms is data that show the target protein binding to the targeted drug. Over the last decade, our team has been developing MSI to characterise some of the most commonly used PM drugs in cancer and inflammatory diseases. Co-localisation of drug and protein target is an extremely challenging but very important task, and requested by the FDA to prove mode-of-drug action. Cases of MM have been studied where a single gene mutation was identified that responded to one of the main therapeutic agents, vemurafenib (Sugihara et al. 2014). In mutant and wild-type patient tumours, a significantly different uptake of the drug in the mutated tumours was observed compared to the wild-type. Expression of the BRAF V600E was demonstrated to coincide with drug binding in areas of BRAF V600E expression (as demonstrated in Fig. 15).

#### Processing MSI data

MSI experiments generate vast numbers of mass spectra, at least one from each of the selected points throughout the tissue area. The combination of this and the high mass resolution of modern instruments leads to data files whose size may easily exceed tens of gigabytes. Pre-processing of the raw data is performed by projecting the spectra onto a common list of peaks or mz-bins. Ion images can then be generated by visualising each peak or bin in a two-dimensional space.

**Fig. 16:**
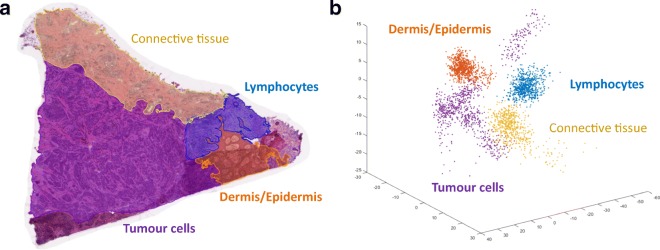
**a** H&E-stained primary tumour from a malignant melanoma patient. Four different regions highlighting the composition of the tissue have been marked by a pathologist. **b** Principal component analysis based on MALDI-MS spectra from each of the four ROIs of the tissue slice. The results were projected on the three first principal components

Users typically query MSI software to determine which analytes co-localise with a known compound, e.g. a drug or drug metabolite. Alternatively, regions of interest (ROIs) corresponding to known structures in the tissue are marked and the question of which peaks are specific to each region is asked, Fig. 16a and b. Finally, there are ways to segment the image in an unsupervised fashion into regions with similar spectral signatures (Suits et al. 2013).

There are multiple ways to perform pre-processing and the choice of method will impact the quality of the ion-images and downstream statistical analysis. The most common approach is to attempt to separate and extract peaks originating from relevant signals, e.g. those originating from structures in the tissue itself, or drugs, from noise and background signals. This is often referred to as peak-picking in the literature. There are numerous algorithms that perform peak-picking and existing MSI software usually implements at least one of these. Alternatively, the spectra can be binned which has the advantage that less data is discarded. Whilst binning may reveal signals that may have been excluded by a peak-picking algorithm, more noisy data is generated. This effectively shifts some of the responsibility from the pre-processing step to later stages (Alexandrov 2012).

Currently, there is no standardised means to perform these tasks. Each instrument vendor provides a software solution with implemented proprietary algorithms. Furthermore, most vendor software only accept their own data format, whereas others also accept the more global imzML format. In addition to proprietary software, there are freeware alternatives such as the R package *Cardinal* (Bemis et al. 2015). These often demand that the user is proficient in programming. In our team, we seek to develop freely available and vendor-agnostic software solutions that aid our researchers and others to interact with the data in an intuitive fashion.

## Outreach programs on cancer awareness

### From innovation to implementation

Decisions that are made concerning patient treatment are highly dependent on reaching a correct diagnosis from pathological evidence, but does modern pathology require more advanced tools? The current situation is that:

1. Diagnosis is observer-dependent, with high inter-observer variability.
2. Due to a multi-step decision process, diagnosis is time-consuming.
3. Sometimes, a definitive diagnosis is not possible.
4. Usually, no prediction of response or outcome can be determined.

Central to the activities of the consortium is the convergence of existing knowledge to arrive at the alternative approach detailed in this review. The aims are to collect patient and tissue-specific molecular information (in the form of big data) and convert this into knowledge upon which serious action can be taken. As discussed above, the key technologies used to analyse 1000s of proteins in 10,000s of cancer types will be merged. A novelty of this approach is the unprecedented scale of analysis. By correlating protein patterns with genomics, pathology and patient (clinical) outcome, rapid and improved cancer diagnosis is anticipated. Ultimately, a personalised treatment decision process for pathologists and medical staff will ensue and lead to the discovery of new targets for improved treatment regimens.

Thus, the focus of our work is to advance and translate innovative research findings directly into clinical applications. Notwithstanding the impressive advances in recent years and those expected to emerge from this research, a significant hurdle still exists in obtaining relevance through the application of new findings and translating this into benefit for patients. The gap between what a researcher ascertains as beneficial innovation and what clinicians require prior to implementation is still very broad. The reasons for this disparity are partially due to the complexity of the task and the attainment of precise molecular information for each individual and for each cancer type.

It is important to remember that despite worldwide efforts to treat melanoma, many unresolved questions remain. To progress, the intimate collaboration between the community of researchers, clinicians and patients is crucial, as is the inclusion of stakeholders and funding agencies. Gaps in knowledge and subsequent hurdles for healthcare professionals can only be resolved by technological approaches. Thus, our aim is to understand melanoma with key mature technologies at an unprecedented scale of operation. In this way, molecular information on patient tissues will be generated that may at first glance appear to be an overwhelming deluge for many colleagues and patients. Thus, the dilemma faced is that a community that can absorb new molecular information and readily apply this to patients or research models does not yet exist. The greatest challenges that lie ahead may not only be in generating new information, but also in aiding clinicians and patients to understand this information in the context of the disease. Patients are important decision-makers in this process and therefore need to understand the information that is generated and the relevance thereof. Important issues for communities are to consider mechanisms for sharing and reuse. How (patient) data will be regulated is one of our immediate challenges.

In summary, merging pathology with our understanding of the location of proteins, the chemical structures of proteins and the pathways where these proteins are functionally active will lead to new and more precise information for each individual and for each cancer type. The potential success of the work described in this review, however, will lie fallow unless successfully translated into information that can be interpreted by humans. To advance from innovative science to implementation in patients through meaningful information requires the concerted efforts of scientists, clinicians and the community. The consortium welcomes and encourages such collaborations.

**Fig. 17:**
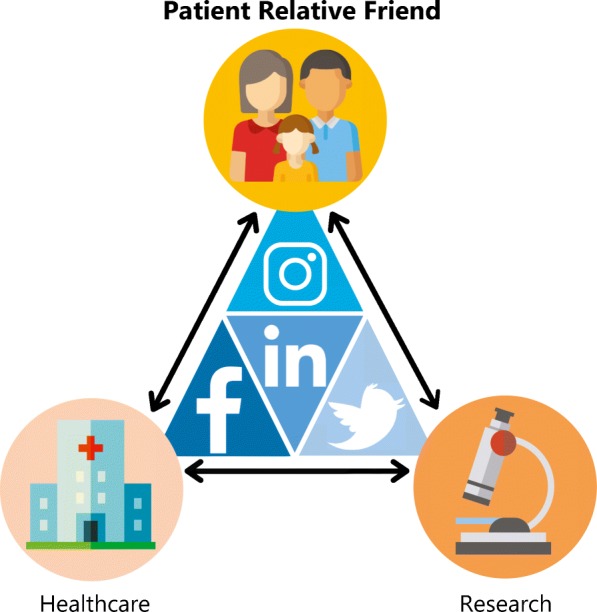
Find the European Cancer Moonshot Lund Center on these social media services. (Facebook, Twitter, Instagram, LinkedIn)

At the European Cancer Moonshot Lund Center, every aspect of the battle against cancer is undertaken with the utmost seriousness. Part of that battle is to increase awareness of the global fight against cancer, and to attract attention to the exceptional research performed by our top scientists and staff—the foot soldiers that tirelessly fight on a daily basis in the war against this relentless disease. To expedite our efforts, the European Cancer Moonshot program has empowered our communications team to boost our visibility and increase awareness of the cooperative research being undertaken with our global partners. This has been achieved through social media platforms of Facebook, Instagram, and Twitter (Fig. 17). Social media are used daily by billions of people and enable organisations such as ours to rapidly and cost-effectively reach a massive and diverse audience that otherwise would be difficult through other more conventional media channels.

**Table 1:** Melanoma types

Type	Age group	Ethnicity	Location	Main cell type	Sun damage	BRAF	NRAS	KIT	NF1	MET	GNA11	GNAQ	NFKBIE
Nodular	Middle adult life	Usually Whites	Any	Epithelioid	Occasionally	20	20	2	2	2	0	0	0
Acro-lentiginous	Late adult life	All races	Palms, soles, subungual	Dendritic	Absent	15	15	20	2	5	0	0	0
Superficial spreading	Middle adult life	Usually Whites	Any	Epithelioid	Occasionally	40	10	2	2	2	0	0	0
Lentigo malignant	Late adult life	Only Whites	Sun-damaged skin	Dendritic	Present	10	10	2	2	2	0	0	0
Desmoplastic	Late adult life	Usually Whites	Sun-damaged skin	Spindle	Present	10	1	4	5	5	0	0	10
Mucosal	Middle adult life	All races	Mucosa	Epithelioid/dendritic	Absent	5	15	20	3	2	0	0	0
Uveal	Middle adult life	Usually Whites	Eye	Epithelioid	Absent	1	1	1	1	1	55	30	0
Spitzoid	Young/early adult life	All races	Any (mainly extremities, head and neck)	Epitheloid/spindle cell	Occasionally	+ in case of BAP1 loss	HRAS	–	–	–	–	–	–

When utilised correctly, social media can aid our organisation in engaging stakeholders, supporters and other research projects, capturing not only global attention but also increasing awareness of cancer and the diligent work of our dedicated team. At the European Cancer Moonshot Lund Center, the most important of all stakeholders are the patients, relatives and friends who are all affected by cancer to differing degrees. With effective communication of our values and continued outreach, many likeminded people will be motivated to become associated with our organisation and supportive of our goals. From our outreach efforts on social media where focus centres on patients, relatives and friends, a community of supporters continues to grow. By raising awareness at the recent European Cancer Moonshot Lund Center symposium, inter-connectivity between the University of Amsterdam, Szeged Medical University, Semmelweis Medical Center and the Cedars Sinai Hospital in Los Angeles has built a common platform to provide value to the patients. Details of these developments can be followed via posted videos of the symposium (https://www.youtube.com/channel/UCjNZ6FYdzKqTrD4VCSnXgAA).

Through this community, our vision is not only actively communicated, but also expedited via direct communication with the organisations and people that our centre and research want to reach. In doing this, not only is our influence increased, but also our credibility. Consequently, we will be better equipped to achieve the goals decreed by the European Cancer Moonshot Lund Center. With patient-, relative- and friend-focused social media outreach programs, the daily impact on the cancer research field will be augmented and enhanced to further our cause.

## Conclusions

Modern science is changing the paradigm of how disease is diagnosed and treated in both a local and global setting of healthcare. Activities in translational science are streamlining the process of drug discovery, and personalised medicine approaches are delivering more effective care. Developing our understanding of disease mechanisms will advance modalities for measuring biomarkers within disease pathways, and clinical proteomics will play an important role in delivering these measurements. Today, however, there is still very little information available to scientifically evaluate drug distribution patterns with an associated biological effect following drug treatment and the impact of medicine on pathology. The development of new analytical tools that can quantitate drugs and metabolites thereof that are delivered at pharmacological doses directly to the site of drug action would provide invaluable data for interpreting the results of drug studies. Drug concentration evaluations are already an integral part of comprehensive safety studies of drug candidates that are a prerequisite to the clinical approval. Drug imaging by mass spectrometry is currently the only available technology that can accurately measure unlabelled drugs in a pharmaceutically administered form. To the best of our knowledge, the results presented here are the first to demonstrate that the interfacing of imaging by mass spectrometry with histopathology is a versatile and simple method to examine drug pharmacokinetic attributes. By extension, this combination further enables the elucidation of the mechanisms-of-drug action at the local in vivo site of intended effect. The constant developments and improvements in mass spectrometry and instrumental platforms will ensure continued delivery of new knowledge on the changes that occur in the histological microenvironment and the subsequent response to drug ‘substances’.

## Abbreviations

ALM: acral lentiginous melanoma
CNN: convolutional neural networks
COPD: chronic obstructive pulmonary disease
DDA: data-dependent acquisition
DIA: data-independent acquisition
DNA: deoxyribonucleic acid
EGFR: epidermal growth factor receptor
FDA: Food and Drug Administration
IMAC: immobilised-metal ion chromatography
LMM: lentigo maligna melanoma
MAKP: mitogen-activated kinase pathway
MetM: metastatic melanoma
MM: malignant melanoma
MS: mass spectrometry
MSI: mass spectrometry imaging
NM: nodular melanoma
NSCLC: non-small-cell lung cancer
PET: positron emission tomography
PLS-Cox: partial least squares–Cox regression
PTM: post-translational-modification
RNA: ribonucleic acid
RPLC: reversed-phase liquid chromatography
SLNB: sentinel lymph node biopsy
SSM: superficial spreading melanoma
TCGA: Tumor Genome Atlas consortium
TILs: tumour-infiltrating lymphocytes
TKIs: tyrosine kinase inhibitors
TMT: tandem mass tag
